# Identification and tracking of HTLV-1–infected T cell clones in virus-associated neurologic disease

**DOI:** 10.1172/jci.insight.167422

**Published:** 2023-04-10

**Authors:** Satoshi Nozuma, Eiji Matsuura, Masakazu Tanaka, Daisuke Kodama, Toshio Matsuzaki, Akiko Yoshimura, Yusuke Sakiyama, Shingo Nakahata, Kazuhiro Morishita, Yoshimi Enose-Akahata, Steven Jacoboson, Ryuji Kubota, Hiroshi Takashima

**Affiliations:** 1 Department of Neurology and Geriatrics, Kagoshima University Graduate School of Medical and Dental Sciences, Kagoshima, Japan.; 2 Division of Neuroimmunology, Joint Research Center for Human Retrovirus Infection, and; 3 Division of HTLV-1/ATL Carcinogenesis and Therapeutics, Joint Research Center for Human Retrovirus Infection, Kagoshima University, Kagoshima, Japan.; 4 Project for Advanced Medical Research and Development, Project Research Division, Frontier Science Research Center, University of Miyazaki, Miyazaki, Japan.; 5 Viral Immunology Section, Neuroimmunology Branch, National Institute of Neurological Disorder and Stroke, NIH, Bethesda, Maryland, USA.

**Keywords:** Inflammation, Virology, Neurological disorders, T cell receptor, T cells

## Abstract

Human T lymphotropic virus type 1–assoicated (HTLV-1–associated) myelopathy/tropical spastic paraparesis (HAM/TSP) is a neuroinflammatory disease caused by the persistent proliferation of HTLV-1–infected T cells. Here, we performed a T cell receptor (TCR) repertoire analysis focused on HTLV-1–infected cells to identify and track the infected T cell clones that are preserved in patients with HAM/TSP and migrate to the CNS. TCRβ repertoire analysis revealed higher clonal expansion in HTLV-1–infected cells compared with noninfected cells from patients with HAM/TSP and asymptomatic carriers (ACs). TCR clonality in HTLV-1–infected cells was similar in patients with HAM/TSP and ACs. Longitudinal analysis showed that the TCR repertoire signature in HTLV-1–infected cells remained stable, and highly expanded infected clones were preserved within each patient with HAM/TSP over years. Expanded HTLV-1–infected clones revealed different distributions between cerebrospinal fluid (CSF) and peripheral blood and were enriched in the CSF of patients with HAM/TSP. Cluster analysis showed similarity in TCRβ sequences in HTLV-1–infected cells, suggesting that they proliferate after common antigen stimulation. Our results indicate that exploring TCR repertoires of HTLV-1–infected cells can elucidate individual clonal dynamics and identify potential pathogenic clones expanded in the CNS.

## Graphical abstract

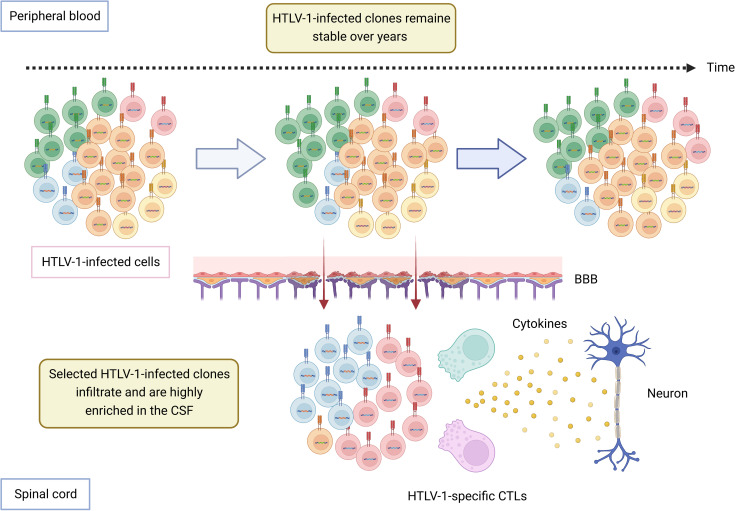

## Introduction

Human T lymphotropic virus type 1 (HTLV-1) infects 5–10 million people worldwide. While most individuals with HTLV-1 infection remain asymptomatic carriers (ACs), approximately 2%–5% develop adult T cell leukemia/lymphoma (ATLL) (1) and another 0.25%–3.8% develop HTLV-1–associated myelopathy/tropical spastic paraparesis (HAM/TSP) (2, 3). HAM/TSP is a neuroinflammatory disease of the CNS, particularly in the spinal cord, presenting with progressive spastic paraparesis and bladder dysfunction. HTLV-1 mainly infects CD4^+^ T cells and is integrated into the host DNA as a provirus. Clonal proliferation of HTLV-1–infected cells is crucial for disease development. Indeed, patients with HAM/TSP have a higher proviral load (PVL) in the cerebrospinal fluid (CSF) as well as in the peripheral blood (4–6), and infected cells aggregate in the spinal cord lesion (7, 8). These HTLV-1–infected T cells express viral proteins that lead to the activation and expansion of HTLV-1–specific CD8^+^ T lymphocytes (CTLs) (9, 10), which subsequently produce proinflammatory cytokines and chemokines and may cause bystander damage to neurons and glial cells in patients with HAM/TSP.

The T cell response to a wide variety of antigens depends on a large repertoire of unique T cell receptors (TCR). TCR diversity is generated through random recombination of genomic variable (V), diversity (D), and joining (J) segments accompanied by insertions and deletions of nucleotides at the joining regions (11). The TCR repertoire is not a static entity, even in healthy persons; healthy individuals may have infections, allergic reactions, and different ages, which can temporarily cause a perturbed TCR repertoire (12). Immune responses to antigen encounter induce activation and clonal expansion of T cells, leading to narrow or skewed TCR repertoire and the emergence of public clonotypes in infection or autoimmune disorders (13, 14). Malignant diseases including leukemia and lymphoproliferative disorders are associated with a monoclonal or oligoclonal expansion of TCR clonotypes derived from cancer cells, and this association allows the detection of minimal residual disease and is used for diagnosis and monitoring of malignancies (15, 16). TCR repertoire analysis by identifying an antigen-specific response, monitoring TCR clonality, and tracking specific TCR clonotypes provides an understanding of T cell–mediated diseases and is a promising biomarker for diagnosis and monitoring of diseases.

In patients with HAM/TSP, several studies have demonstrated skewed TCR repertoires and clonal expansions in CD4^+^ and CD8^+^ T cells as well as in peripheral blood lymphocytes (17–20). TCR repertoire analysis with the high-throughput sequencing (HTS) technique has identified TCR repertoire signatures in HTLV-1–specific CTLs, and these potential pathogenic CTL clones are enriched in the CSF (21). HTLV-1 propagates infection by cell-to-cell contacts and clonal proliferation of infected cells, which are associated with the development of ATLL and HAM/TSP. ATLL cells are characterized by the presence of monoclonal or oligoclonal T cell expansions (22, 23). The oligoclonal proliferation of HTLV-1–infected cells has previously been considered to be the pathogenesis of HAM/TSP; however, integration site analysis of HTLV-1 failed to show a characteristic pattern of clonality in peripheral blood mononuclear cells (PBMCs) of patients with HAM/TSP (24). Because circulating HTLV-1–infected T cells migrate across the blood-brain barrier and cause neuroinflammation, exploring a TCR repertoire in HTLV-1–infected T cells will help elucidate the pathogenesis of HAM/TSP.

The purpose of this study is to examine whether HTLV-1–infected T cells remain within individual patients with HAM/TSP at the clonal level by TCR repertoire analysis. Longitudinal analysis showed that highly expanded clones in HTLV-1–infected T cells were preserved over time. Additionally, we also aimed to investigate whether the HTLV-1–infected cell clones migrate passively from the peripheral blood or proliferate compartmentally in the CNS and TCR repertoire analysis in paired HTLV-1–infected T cells and CSF cells that were identified putatively as pathogenic HTLV-1–infected T cell clones that were expanded and enriched in the CNS.

## Results

### TCR repertoires show high expansion of HTLV-1–infected T cells in patients with HAM/TSP and ACs.

**Figure 1:**
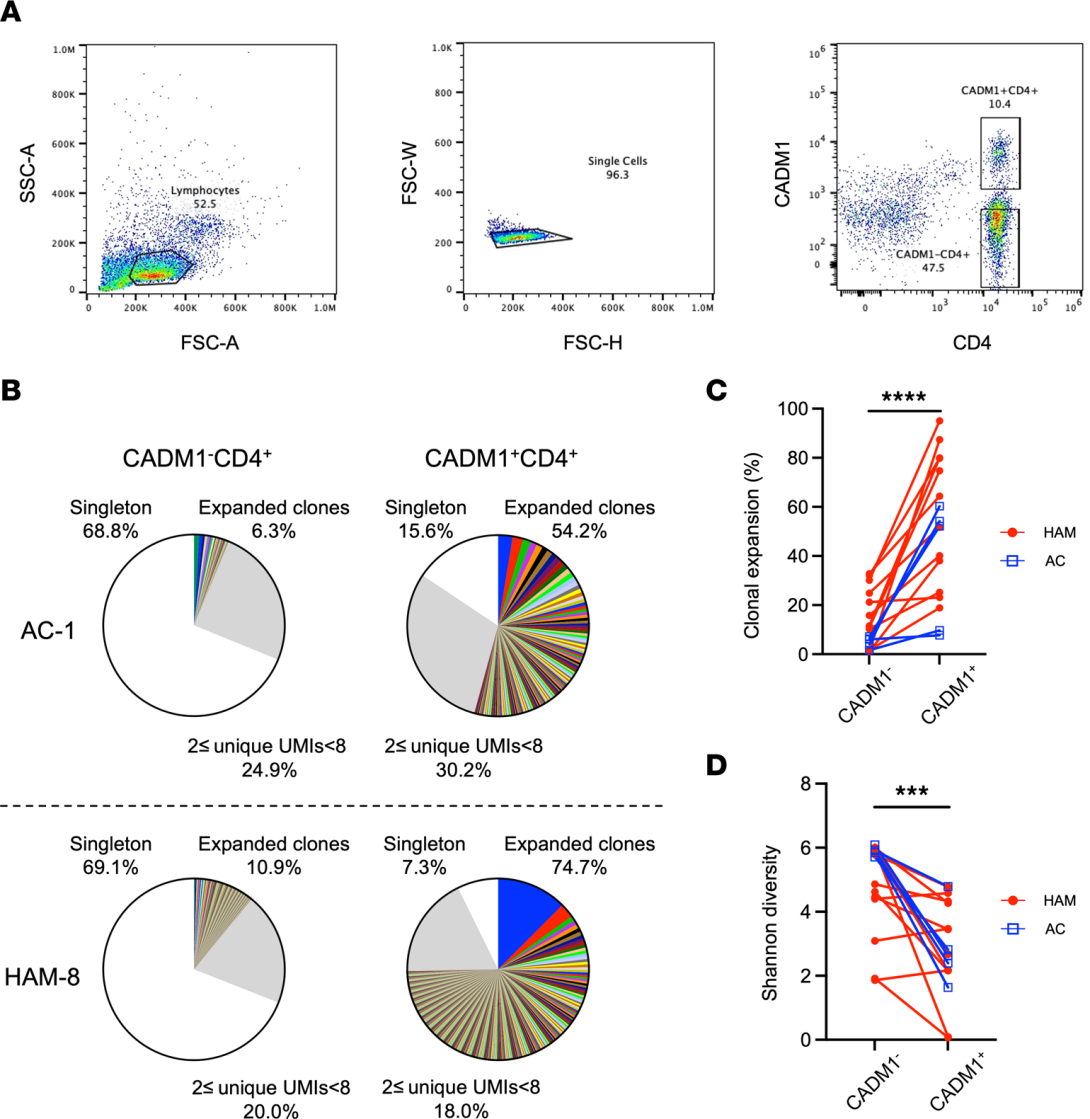
Clonal expansion of TCRβ repertoire in HTLV-1–infected cells of patients with HAM/TSP and ACs. (**A**) Gating strategy for flow cytometry sorting of CADM1^+^CD4^+^ and CADM1^–^CD4^+^ T cells from PBMCs samples. (**B**) Representative examples of T cell clonal expansion in CADM1^–^CD4^+^ and CADM1^+^CD4^+^ T cells of an AC (AC-1) and a HAM/TSP patient (HAM8). Clonal expansion of TCRβ repertoire is classified by the frequencies of clones ≥ 8 UMIs (color wedges), clones with 2 ≤ UMIs < 8 (gray), and singletons (white). In the group of expanded clones, each wedge exhibits a unique clonotype with a defined CDR3 sequence, and the identical clones shared by CADM1^–^CD4^+^ and CADM1^+^CD4^+^ T cells in each individual are depicted in the same color. (**C** and **D**) Comparison of T cell clonal expansion by frequency of clones ≥ 8 UMIs (**C**) and Shannon diversity (**D**) between CADM1^–^CD4^+^ and CADM1^+^CD4^+^ T cells from ACs (*n* = 5) and patients with HAM/TSP (*n* = 12) using Wilcoxon signed-rank test. ***P < 0.001, ****P < 0.0001.

To investigate the TCR repertoire in HTLV-1–infected T cells, we performed HTS of the TCRβ chain from 17 subjects consisting of 12 patients with HAM/TSP and 5 ACs. The clinical characteristics of the subjects are listed in Table 1. The median (interquartile range [IQR]) age of patients with HAM/TSP and ACs was 67.5 (54.8–76.5) and 55.0 (37.0–66.0) years, respectively (no statistically significant differences). As previously reported (25), the median HTLV-1 PVL in PBMCs in patients with HAM/TSP was 12.0% (5.4%–17.7%), which was significantly higher than that in ACs (2.2% [1.4%–3.4%]; *P* < 0.001; Table 1). The median disease duration was 9.0 years (5.5–17.0 years) in patients with HAM/TSP. Two patients were treated with 5 mg of prednisolone, and the other 2 patients were treated with antispastic drugs. HTLV-1–infected cells were sorted using the CADM1 antibody, which is the most common surface marker for identifying HTLV-1–infected cells (26, 27) (Figure 1A). The frequency of CADM1^+^CD4^+^ T cells significantly correlated with the PVL in PBMCs as previously reported (27) (*P* < 0.01; Supplemental Figure 1A; supplemental material available online with this article; https://doi.org/10.1172/jci.insight.167422DS1). Patients with HAM/TSP had a significantly higher frequency of CADM1^+^CD4^+^ T cells, compared with ACs (*P* < 0.05; Supplemental Figure 1B), consistent with the higher PVL detection in patients with HAM/TSP.

To confirm the skewed TCR repertoire in HTLV-1–infected T cells, we first compared the TCRβ repertoires between sorted CADM1^+^CD4^+^ T cells and CADM1^–^CD4^+^ T cells. The summary of the TCRβ sequence data including the number of total HTS sequences, total unique molecular identifies (UMIs), total unique TCRβ clonotypes, and Shannon diversity (28) from both patients with HAM/TSP and ACs are shown in Supplemental Table 1. On average, 595,859 and 510,305 sequence reads were generated from the CADM1^+^CD4^+^ and the CADM1^–^CD4^+^ T cells, respectively (Supplemental Figure 1C and Supplemental Table 1). The number of total unique TCRβ clonotypes tended to be lower in CADM1^+^CD4^+^ T cells compared with CADM1^–^CD4^+^ T cells (Supplemental Figure 1D and Supplemental Table 1), and the number of unique TCRβ clonotypes was significantly lower in CADM1^+^CD4^+^ compared with CADM1^–^CD4^+^ T cells (*P* < 0.0001; Supplemental Figure 1E and Supplemental Table 1). The TCRβ clonal size was divided into 3 categories based on the number of UMIs: (a) singletons (1 UMI); (b) clones with 2 ≤ UMIs < 8; and (c) expanded clones with ≥ 8 UMIs, as previously described (20, 21) (see Methods section for classification criteria). Representative examples of expanded TCRβ clones in a patient with HAM/TSP and an AC are depicted in Figure 1B. In this pie chart, according to our definition of TCR clonal size, expanded clones with ≥ 8 UMIs are visualized as colored wedges, while clones with 2 ≤ UMIs < 8 and singletons are exhibited in the gray area and in the white area, respectively. Each colored wedge indicates a unique clonotype with a defined complementarity-determining region 3 (CDR3) sequence. The frequency of expanded clones varied among subjects both in CADM1^+^CD4^+^ and CADM1^–^CD4^+^ cells (Figure 1B and Supplemental Figure 2). While polyclonal TCR repertoires were observed in most samples, oligoclonal expansions were detected in CADM1^+^CD4^+^ T cells and in CADM1^–^CD4^+^ T cells (Figure 1B and Supplemental Figure 2). Quantification of these results is shown in Figure 1C, where each point indicates the frequency of T cell clonal expansion in CADM1^+^CD4^+^ and CADM1^–^CD4^+^ cells of patients with HAM/TSP and ACs. The CADM1^+^CD4^+^ populations had significantly higher clonal expansions, compared with CADM1^–^CD4^+^ T cells (*P* < 0.0001; Figure 1C). Additionally, estimation of the TCR clone diversity using the Shannon diversity index, which normalizes the number of unique T cell clonotypes via resampling to the size of the smallest data set of sorted CADM1^+^CD4^+^ or CADM1^–^CD4^+^ cells applying the Shannon entropy algorithm (28), showed a significantly lower diversity in CADM1^+^CD4^+^ compared with CADM1^–^CD4^+^ T cells (*P* < 0.001; Figure 1D). These results indicate that the clonal expansion of the TCR repertoire is high in HTLV-1–infected cells of patients with HAM/TSP and ACs, compared with noninfected cells.

### TCR clonality in HTLV-1–infected cells is not different between patients with HAM/TSP and ACs.

**Figure 2:**
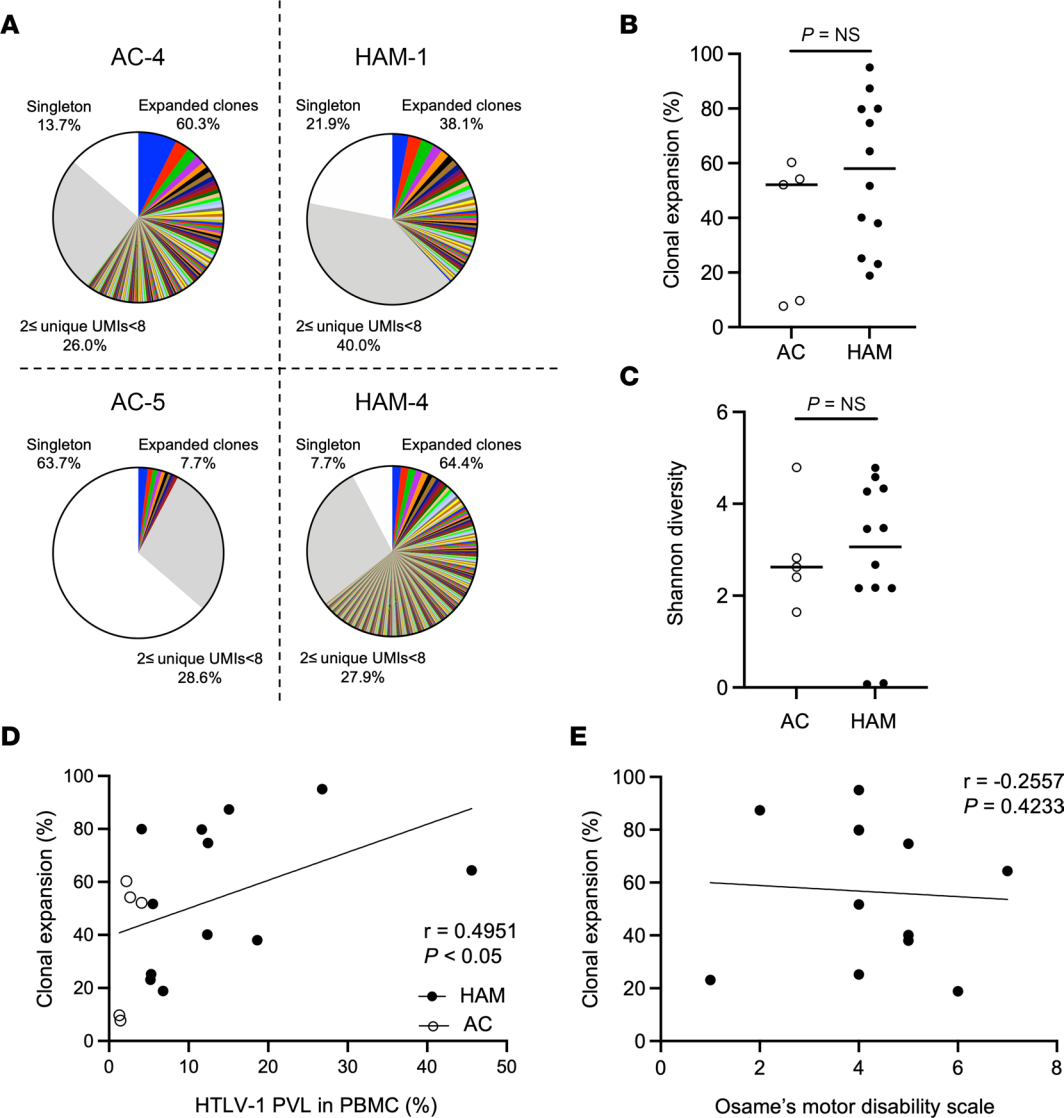
Comparison of TCRβ clonal repertoire analysis of CADM1^+^CD4^+^ T cells from patients with HAM/TSP and ACs. (**A**) Representative analysis of the clonal expansion of TCR repertoire in CADM1^+^CD4^+^ cells from ACs and patients with HAM/TSP. (**B**) Comparison of T cell clonal expansion between ACs (*n* = 5) and patients with HAM/TSP (*n* = 12) using Wilcoxon signed-rank test. The horizontal bar indicates the median. (**C**) Comparison of Shannon diversity between ACs (*n* = 5) and patients with HAM/TSP (*n* = 12) using Wilcoxon signed-rank test. The horizontal bar indicates the median. (**D**) Correlation of clonal expansion in CADM1^+^CD4^+^ T cells with HTLV-1 PVL in PBMCs from ACs (opened shapes; *n* = 5) and patients with HAM/TSP (closed shapes; *n* = 12) using Spearman’s rank correlation test. (**E**) Correlation of clonal expansion in CADM1^+^CD4^+^ T cells with OMDS of patients with HAM/TSP (*n* = 12) using Spearman’s rank correlation test.

To examine whether the clonality in HTLV-1–infected cells differs between disease states, we compared the TCRβ repertoire in HTLV-1–infected T cells between patients with HAM/TSP and ACs. While the frequency of expanded clones varied between participants, most participants showed polyclonal clones, but some participants had oligoclonal clones in HTLV-1–infected cells in ACs as well as in patients with HAM/TSP (Figure 2A and Supplemental Figure 2). When the frequency of clonal expansion was examined, there was no significant difference between patients with HAM/TSP and ACs (Figure 2B). The Shannon diversity index also did not differ between disease states (Figure 2C and Supplemental Table 1).

While our results show that the frequency of clonal expansion and Shannon diversity in HTLV-1–infected cells did not differ between patients with HAM/TSP and ACs, the patients with HAM/TSP had a higher PVL compared with ACs (Table 1). As shown in Figure 2D, the frequency of clonal expansion in HTLV-1–infected T cells significantly correlated with the HTLV-1 PVL (*P* < 0.05). When examining the association of clinical states, there was no correlation between the frequency of clonal expansion and the Osame motor disability score (OMDS; Figure 2E). Collectively, the results reveal that the degree of clonal expansion in HTLV-1–infected cells correlated with the HTLV-1 PVL; however, the frequency of clonal expansion was not different between patients with HAM/TSP and ACs.

### TCRβ repertoire in HTLV-1–infected T cells is preserved in individual patients with HAM/TSP over years.

**Figure 3:**
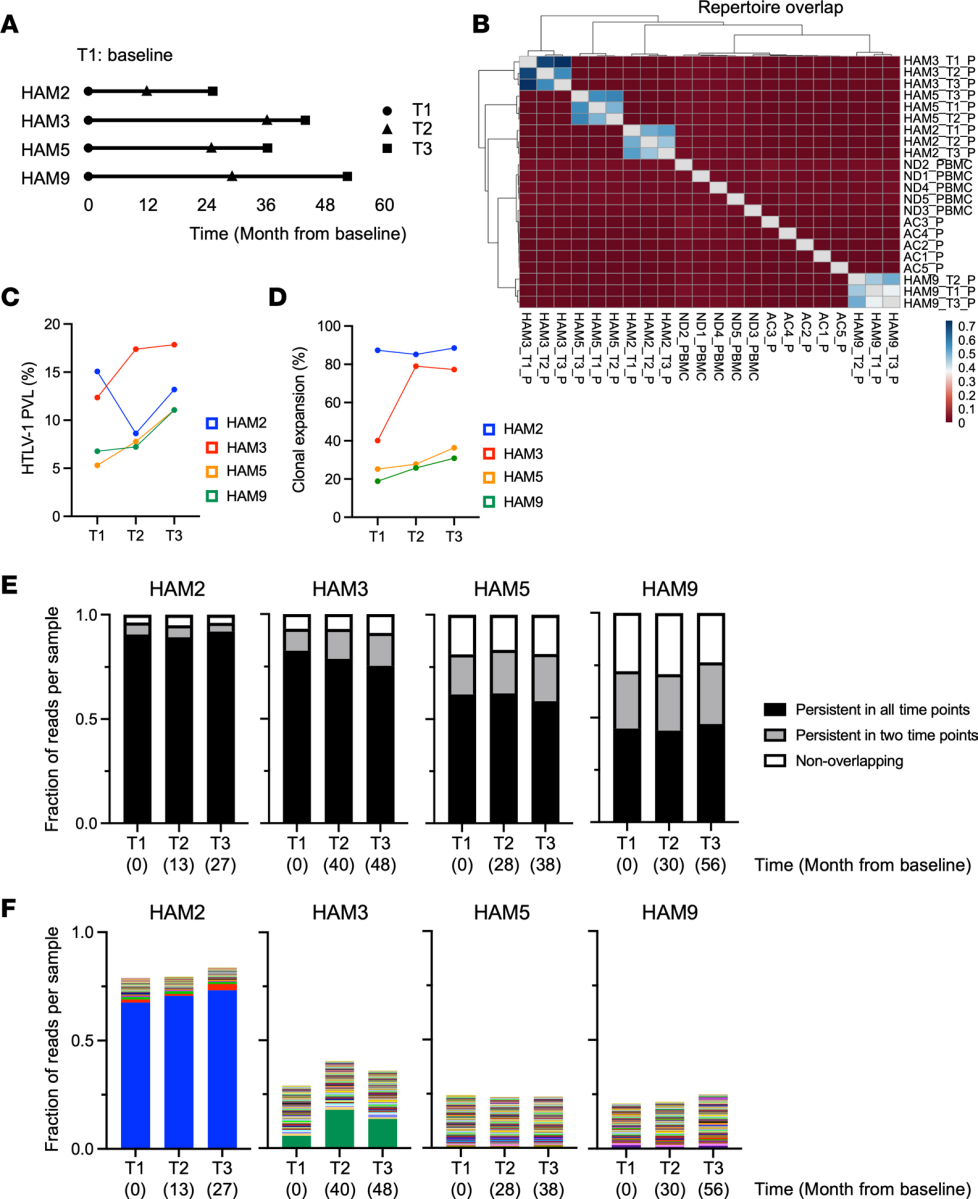
Longitudinal analysis of the TCRβ repertoire in HTLV-1–infected T cells from patients with HAM/TSP. (**A**) Scheme of longitudinal sampling. The duration from T1 to T3 is more than 2 years, and each time point represents a period of at least 8 months. (**B**) A heatmap analysis represents the repertoire overlap of TCRβ clonotypes shared among 3 longitudinal samples from 4 patients with HAM/TSP, 5ACs, and 5 normal donors using the overlap coefficient, a normalized measure of overlap similarity. (**C** and **D**) Longitudinal HTLV-1 PVL in PBMCs (**C**) and clonal expansion of HTLV-1–infected T cells (**D**) from 4 patients with HAM/TSP across 3 time points. (**E**) Dynamics of TCRβ clones in HTLV-1–infected cells. Persistent clones were investigated by the frequencies of the sequences persistent across 3 time points (black), at least 2 time points (gray), and nonoverlapping (white). (**F**) Tracking the 100 most dominant clonotypes shared across all time points in each HAM/TSP patient. The identical clones shared across all time points in each individual are depicted in the same color.

While a high HTLV-1 PVL is the main risk factor for developing HAM/TSP, the HTLV-1 PVL in PBMCs is considered stable over time within each HTLV-1–infected person, including within patients with HAM/TSP. To examine the clonal dynamics in HTLV-1–infected T cells, we analyzed the TCRβ repertoire in 4 patients with HAM/TSP across 3 time points (T1–T3). The scheme of the longitudinal analysis is shown in Figure 3A. The duration from T1 to T3 was more than 2 years, and each time point occurred over a period of at least 8 months. HAM2 was treated with 5 mg of prednisolone and HAM5 with dantrolene, and the treatment had not changed during the observation period (Table 1). We first examined whether there are public TCRβ clonotypes in HTLV-1–infected T cells across patients with HAM/TSP. A heatmap analysis shows the quantification of the overlap in TCRβ repertoire within samples using the overlap coefficient, which is a normalized measure of overlap similarity and is defined as the size of the intersection divided by the smaller size of the 2 sets (Figure 3B). While many TCR clonotypes were shared within each patient, the overlap was extremely limited among samples from different patients. These results demonstrate that the TCR repertoire is unique to individual patients even in HTLV-1–infected T cells from patients with HAM/TSP.

Next, we examined whether the clonal expansion in HTLV-1–infected T cells correlated with the HTLV-1 PVL longitudinally (Figure 3, C and D, and Supplemental Figure 3). A previous report has shown a correlation between T cell clonal expansion in PBMCs and PVL in patients with HAM/TSP over 2 years (20). While 3 patients with HAM/TSP (HAM3, HAM5, and HAM9) showed that the degree of clonal T cell expansion was associated with HTLV-1 PVL, another patient (HAM2) showed a transient decrease in clonal T cell expansion that was associated with decreased PVL at T2 (Figure 3, C and D). These longitudinal results are consistent with the positive correlation of PVL with clonal expansion in HTLV-1–infected cells, as shown in Figure 2D.

To examine the dynamics of HTLV-1–infected T cell clones within individual patients, we compared clones divided into 3 groups: (a) persistent in all time points, (b) persistent in 2 time points, and (c) nonoverlapping. Most clones in the HTLV-1–infected T cells persisted, and the proportion of persistent clones was stable in each patient over time (Figure 3E). For example, approximately 90% of the clones were shared in all time points and 95% of clones were shared in 2 time points in HAM2 (Figure 3E). Likewise, approximately 60% of the clones in the infected cells persisted in all time points and 80% of clones persisted in 2 time points in HAM5. Interestingly, the TCR repertoire in the patients with higher expanded clones (e.g., HAM2 and HAM3; Figure 3D) was largely dominated by persistent clones (Figure 3E).

Because highly proliferating HTLV-1–infected T cells are thought to cause inflammation in the host or lead to progression to ATLL cells, we analyzed the dynamics of highly expanded clones within each patient. Longitudinal tracking of the top 100 most abundant clonotypes shared in all time points is shown in Figure 3F. As expected, the proportion of the top 100 clones did not change in each patient during the observation period. Moreover, the dominant clones, which are represented by the blue box in HAM2 and the green box in HAM3, remained stable over time (Figure 3F). These results indicate that the TCR repertoire in HTLV-1–infected T cells remains unchanged and that highly expanded clones are preserved within individual patients with HAM/TSP over the years.

### Expanded HTLV-1–infected clones show different distributions between the CSF and peripheral blood and are enriched in the CSF of patients with HAM/TSP.

**Figure 4:**
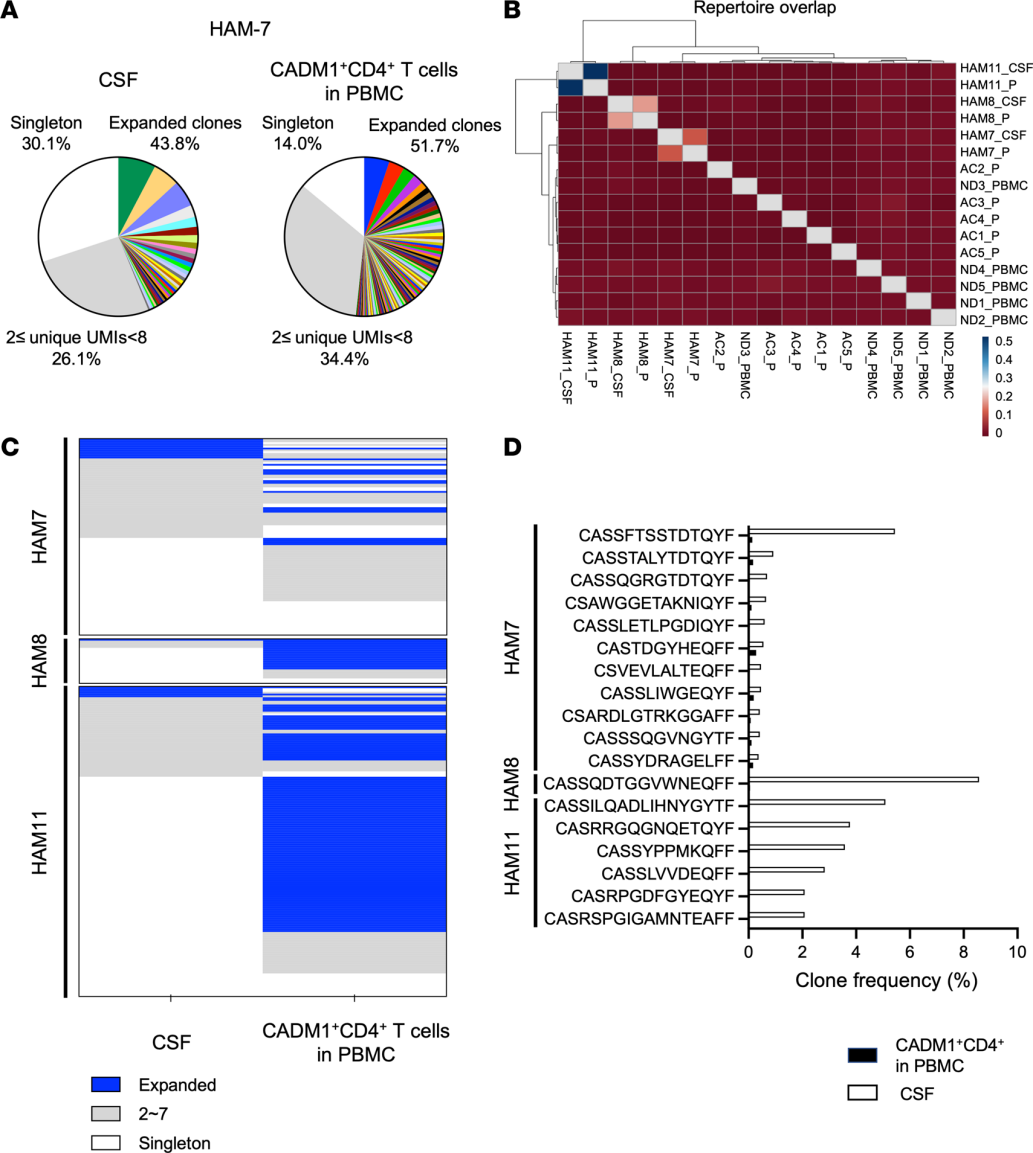
Differential distribution and enrichment of expanded HTLV-1–infected clones in the CSF compared with the peripheral blood of patients with HAM/TSP. (**A**) Pie chart representations of T cell clonal expansion in CSF cells and CADM1^+^CD4^+^ cells of a HAM/TSP patient (HAM7). (**B**) A heatmap analysis exhibiting an overlap of TCRβ clonotypes among 3 paired CSF and CADM1^+^CD4^+^ cells from 3 patients with HAM/TSP, 5 ACs, and 5 normal donors using the overlap coefficient. (**C**) Heatmap of shared clonotypes in CSF cells and CADM1^+^CD4^+^ cells in PBMCs from 3 patients with HAM/TSP. Each row slice represents the shared TCRβ clonotypes between CSF cells and CADM1^+^CD4^+^ cells in PBMCs of each HAM/TSP patient. Expanded clonotypes with ≥ 8 UMIs are shown in the blue area, while clonotypes with 2 ≤ UMIs < 8 or singletons are visualized in the gray area and in the white area, respectively. For example, of 11 expanded clones shown in blue slices in the CSF of HAM7, only 1 clonotype (9.1%) was shared with the expanded clones in the peripheral CADM^+^CD4^+^ T cells and 10 clonotypes (90.9%) were shared with singleton (white) or 2 ≤ UMIs < 8 (gray). Conversely, of 16 expanded clones shown in blue slices in peripheral CADM1^+^CD4^+^ T cells of HAM7, only 1 clonotype (6.3%) was shared with the expanded clones in CSF cells and 15 clonotypes (93.7%) were shared with singleton or 2 ≤UMIs < 8. (**D**) Frequencies of 18 TCRβ expanded clonotypes in the CSF compared with peripheral CADM1^+^CD4^+^ cells in 3 patients with HAM/TSP.

Circulating HTLV-1–infected T cells have been shown to infiltrate the spinal cord across the blood-brain barrier and are thought to initiate an inflammatory response against the virus and/or components of the CNS. Because HTLV-1–infected clones expanded in the CNS are considered pathogenic, we wondered whether the expanded clones migrate passively from the peripheral blood or proliferate compartmentally in the CNS. We compared the TCRβ repertoire in 3 paired CSF cells and CADM1^+^CD4^+^ T cells sorted from PBMCs in patients with HAM/TSP. As previously reported (6), the HTLV-1 PVL in the CSF was higher than that in PBMCs (Table 1). The number of sequence reads, UMIs, and unique TCRβ clonotypes is summarized in Supplemental Table 1. Figure 4A shows a representative example of the frequency of expanded TCRβ clonotypes in paired CSF cells and CADM1^+^CD4^+^ T cells from patient HAM7. Individual variations in clonal expansion were observed in the CSF of patients with HAM/TSP (Figure 4A and Supplemental Figure 4). The same clone shared by the CSF and CADM1^+^CD4^+^ T cells in each patient is visualized by the same color. We first examined whether public clones were detected in both the CSF cells and CADM1^+^CD4^+^ T cells using exact CDR3 sequences; however, a high overlap of TCRβ clonotypes was found within individuals, and a limited overlap was observed between individuals in CSF cells as well as CADM1^+^CD4^+^ T cells of patients with HAM/TSP (Figure 4B).

To examine how HTLV-1–infected T cells infiltrating the CSF are represented in the peripheral blood, we compared the TCRβ sequences shared between paired CSF cells and CADM1^+^CD4^+^ T cells in PBMCs. TCRβ clonotypes shared by CSF and CADM1^+^CD4^+^ T cells in PBMCs from 3 patients with HAM/TSP are shown in Figure 4C and are listed in Supplemental Table 2. Comparison of the distribution of TCRβ clonotypes in the compartments indicated that expanded clonotypes in the CSF cells were not always proliferated in the peripheral CADM1^+^CD4^+^ T cells. For example, of 11 expanded clones depicted in blue in the CSF of HAM7, only 1 clonotype (9.1%) was shared with the expanded clones in the peripheral CADM^+^CD4^+^ T cells, and 10 clonotypes (90.9%) were shared with singleton or 2 ≤ UMIs < 8 (Figure 4C and Supplemental Table 2). Likewise, of 6 expanded clones in the CSF of HAM11, only 2 clonotypes (33.3%) were detected in the expanded clones of CADM1^+^CD4^+^ T cells in PBMCs and 4 clonotypes (66.7%) in singleton or 2 ≤ UMIs < 8 (Figure 4C and Supplemental Table 2). Conversely, of 16 expanded clones depicted in blue in peripheral CADM1^+^CD4^+^ T cells of HAM7, only 1 clonotype (6.3%) was shared with the expanded clones in CSF cells, and 15 clonotypes (93.7%) were shared with singleton or 2 ≤UMIs < 8 (Figure 4C and Supplemental Table 2). These results demonstrate that expanded TCR clonotypes in the CSF cells were not always detected in the clonally expanded pools of the peripheral HTLV-1–infected cells. The frequencies of clonally expanded TCRβ clonotypes in the CSF derived from the peripheral HTLV-1–infected cells are shown in Figure 4D and are listed in Table 2. Comparison of the frequency of the 18 TCRβ sequences shared by peripheral CADM1^+^CD4^+^ T cells and CSF cells from 3 patients with HAM/TSP shows that these clonotypes were more abundant in the CSF compartments compared with the peripheral blood (Figure 4D and Table 2). Collectively, our results indicate that expanded HTLV-1–infected T cells in the CSF cells are not always detected in the clonally expanded pools of the peripheral HTLV-1–infected cells. While putatively pathogenic HTLV-1–infected clones do not just cross the blood-brain barrier passively, selected clones infiltrate and are highly enriched in the CSF compartment.

### TCR clusters can be identified in proliferating HTLV-1–infected T cells.

**Figure 5:**
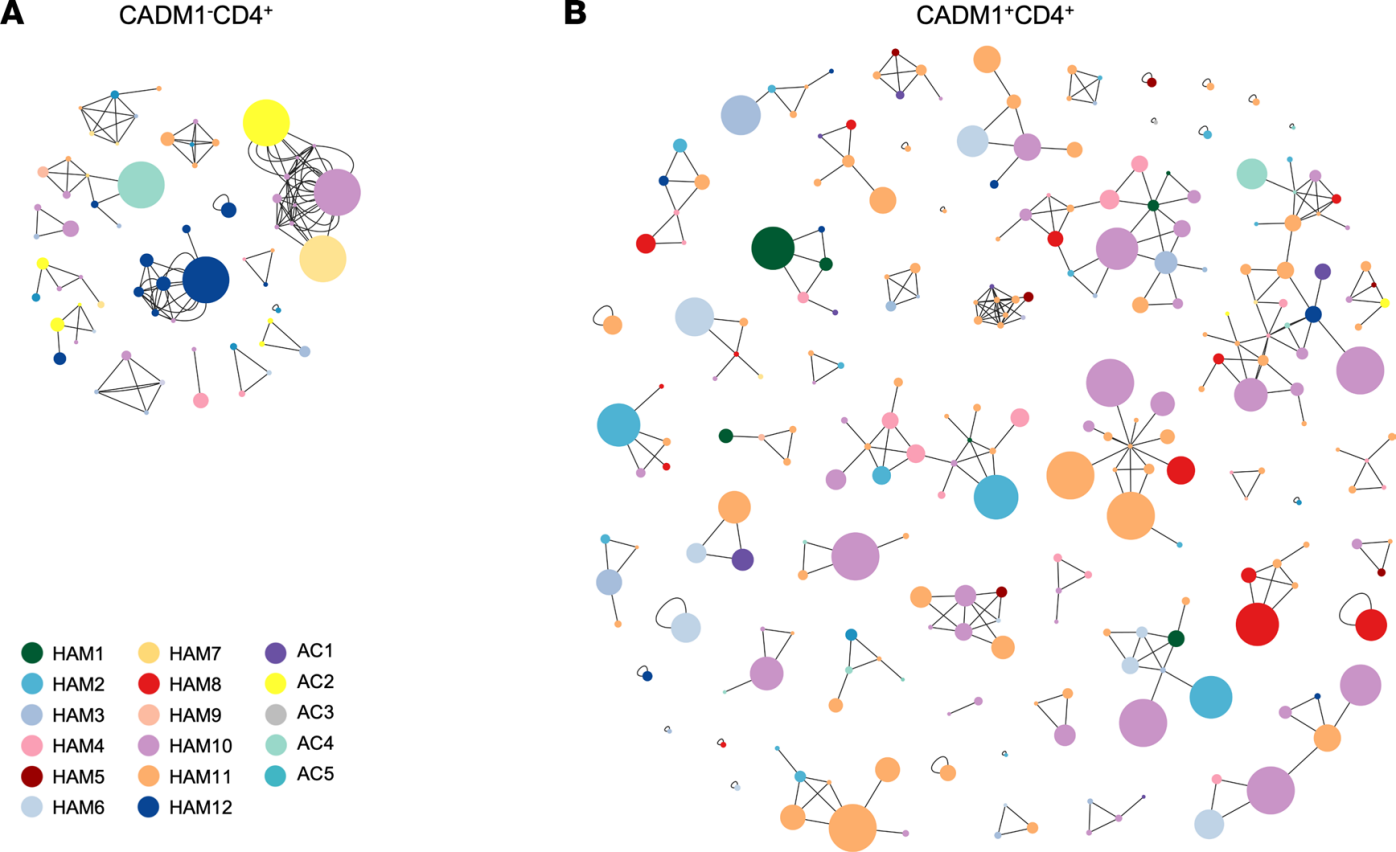
Clustering analysis of similarity in TCRβ sequences in expanded HTLV-1–infected cells of patients with HAM/TSP and ACs. (**A** and **B**) Network plot showing clusters of TCRβ in expanded CADM1^–^CD4^+^ T cells (**A**) and CADM1^+^CD4^+^ T cells (**B**) in PBMCs of patients with HAM/TSP (*n* = 12) and ACs (*n* = 5) using GLIPH2 software. Each node represents a unique CDR3 sequence with node colors based on subjects and node size according to read counts.

The HTLV-1 virus induces the proliferation of infected T cells by expressing viral proteins. However, because CD4^+^ T cells are activated and expanded by antigen stimulation, infected T cells would proliferate in response to common antigens. To examine whether clonal expansion by antigen stimulation is detected in proliferating HTLV-1–infected T cells, TCR clustering was analyzed in the expanded clonotypes (≥ 8 UMIs) of paired CADM1^–^CD4^+^ T cells and CADM1^+^CD4^+^ T cells sorted from PBMCs of patients with HAM/TSP and ACs using the GLIPH2 algorithm. GLIPH2 is a software that analyzes antigen-specific clustering by shared structural features (29). We first examined clustering using all the TCRβ clonotypes, and clonotypes from CADM1^+^CD4^+^ T cells had a higher frequency of clusters than those from CADM1^–^CD4^+^ T cells (6.6 versus 0.2 clusters every 1,000 TCRβ input clonotypes; Supplemental Table 3). For TCR clustering analysis in expanded clonotypes, a total of 2,349 and 5,590 clonotypes from CADM1^–^CD4^+^ T cells and CADM1^+^CD4^+^ T cells, respectively, were used as input for GLIPH2. Figure 5, A and B, shows a network of TCRβ sequences based on clustering analysis by GLIPH2, and all clusters are shown in Supplemental Table 4. Fifteen clusters of 65 clonotypes were detected in CADM1^–^CD4^+^ cells (Figure 5A), whereas 54 clusters of 244 clonotypes were found in CADM1^+^CD4^+^ T cells (Figure 5B), suggesting that TCR repertoires in HTLV-1–infected cells shared a similar clone structure and connectivity across subjects. These results suggest that a portion of the HTLV-1–infected T cells may clonally proliferate after a common antigen stimulation.

## Discussion

Identification and characterization of pathogenic cells provide a better understanding of the pathophysiology of infections and autoimmune diseases. Because HTLV-1 infects and proliferates T cells, TCR repertoire analysis is an ideal technique to explore the characterization of HTLV-1–infected T cell clones. Longitudinal tracking of proliferated HTLV-1–infected clones may serve as an indicator of disease progression. Additionally, exploring the TCR repertoire signatures in expanded TCR sequences in the CNS may uncover pathological T cell clones that cause inflammation and neural damage in patients with HAM/TSP. In the present study, we analyzed the TCR repertoire targeting HTLV-1–infected T cells and showed highly expanded clones in infected populations. Longitudinal analysis revealed that infected T cell clones were highly stable over years and were largely dominated by clonally expanded clonotypes. The TCRβ repertoire in paired HTLV-1–infected T cells and CSF cells showed clonal expansion and different distribution in the CSF compared with the peripheral blood.

Our longitudinal study shows that the TCR repertoire in HTLV-1–infected clones from patients with HAM/TSP is preserved for years. More than half of the clones survived in the HTLV-1–infected cells, and their proportion remained stable within each HAM/TSP patient over time (Figure 3E). Dynamic changes in the TCR repertoire are a common feature in the response to viral infections. However, patients with untreated HIV infection or people living with HIV on long-term antiretroviral therapy (ART) show significantly less TCR repertoire diversity (30, 31). Additionally, effective ART that reduced HIV viral load and increased CD4^+^ T cell numbers did not recover the overall reduction in the TCR repertoire diversity (32). Our TCR sequence analysis revealed that TCR repertoires were significantly expanded in HTLV-1–infected cells compared with noninfected cells and that the individual’s TCR signature was maintained for years in patients with HAM/TSP. Moreover, highly proliferating clones that were observed in more than 1% of the individual TCR repertoire were identified in the longitudinal study. These highly expanded clones are considered pathogenic clones that can cause inflammation in the host and even progress to ATLL. Indeed, HTLV-1 proviral integration site analysis revealed that patients with HAM/TSP and ACs with highly expanded clones having an oligoclonal or monoclonal pattern developed ATLL (33, 34). Recently, a monoclonal antibody therapy targeting C-C chemokine receptor type 4 (CCR4), which is expressed on HTLV-1–infected cells, was used in a clinical trial with patients with HAM/TSP, showing a reduction in infected cells (35). Though the PVL measurement is widely used for diagnosis, detailed clonal analysis of HTLV-1–infected cells provides a better indicator for monitoring disease states and assessing treatment efficacy of HAM/TSP. TCR repertoire analysis can detect long-lived, proliferating HTLV-1-infected clones and may be a surrogate marker of disease monitoring and treatment response.

The PVL is higher in the CSF than in peripheral blood (5, 6), and accumulation of infected cells has been identified in the spinal cord lesion in patients with HAM/TSP (7, 8). HTLV-1–infected cells produce INF-γ that stimulates astrocytes to secret the chemokine CXCL10, which recruits more CXCR3^+^ T cells — including infected cells — into the CNS, leading to an inflammatory response and neural damage (36). In the present study, we detected shared clones between paired peripheral CADM1^+^CD4^+^ T cells and CSF cells from the same patients with HAM/TSP, suggesting that the shared clones infiltrated into the CSF from the peripheral blood. Additionally, the distribution of expanded TCRβ clonotypes differed between the peripheral blood and the CSF, and the expanded TCRβ clonotypes in the CSF were more enriched than in the peripheral blood. These results revealed that HTLV-1–infected T cells that infiltrated into the CNS do not just transit passively across the blood-brain barrier, whereas selected HTLV-1–infected T cells infiltrate into the CNS from the peripheral blood and clonally expand in the CNS, leading to inflammation and neural damage in patients with HAM/TSP (37). The TCR repertoire signature in the local lesion is closely related to the pathogenesis of the disease. In patients with multiple sclerosis, which is the most common neuroinflammatory disease, EBV-reactive T cell clones are compartmentalized and enriched in the CNS (38, 39). We have previously reported that HTLV-1–specific CTLs were expanded and enriched in the CSF of patients with HAM/TSP (21). In malignancies, the degree of diversity of the TCR repertoire in local cancer tissue is associated with treatment response and disease prognosis (40–42). The entry of pathogenic T and B cells from the peripheral blood and their proliferation and activation in the CNS play major roles in the pathogenesis of neuroinflammatory diseases. Indeed, natalizumab, a treatment that blocks the migration of peripheral lymphocytes into the CNS, has shown efficacy in patients with multiple sclerosis (43, 44). Clarification of the clonal behavior of HTLV-1–infected cells in the CNS may lead to therapeutic development.

Unlike most virus infections, the replication of HTLV-1 is very low, and cell-free viral particles are rarely detected in vivo. Instead, HTLV-1 propagates and persists in the host via de novo cell-to-cell infection and mitosis of the infected cells (45). While mitotic spread is predominant in the chronic phase of HTLV-1 infection, a recent study using computational and mathematical models has estimated that new HTLV-1–infected clones contribute to maintaining infected clones even after the HTLV-1 PVL reaches a steady state (46). In mitotic spread, an infected cell duplicates and splits into 2 identical daughter cells, which carry the same TCR clonotype, whereas de novo infection spreads the virus from infected T cells to uninfected cells, resulting in the cells bearing different TCR clonotypes. Our study shows that increased HTLV-1 PVL correlated with the higher clonal expansion of HTLV-1–infected cells (Figure 2D). Additionally, the longitudinal analysis reveals that increases in HTLV-1 PVL were associated with the elevated clonal expansion of HTLV-1–infected cells in individual patients over time (Figure 3, C and D). These results indicate that the increase in HTLV-1–infected cells is due to an expansion of individual infected cell clones, supporting the involvement of mitotic proliferation rather than de novo infection in the increase in HTLV-1–infected cells during the chronic phase.

A previous study using analysis of the HTLV-1 integration site showed that clonality did not distinguish patients with HAM/TSP from ACs when patients with HAM/TSP and ACs with similar PVL were compared (24). In the current study, clonal expansion of the TCRβ repertoire in HTLV-1–infected T cells was not different between HAM/TSP and ACs despite the higher PVL observed in patients with HAM/TSP. Furthermore, patients with HAM/TSP with a higher PVL showed equal or lower clonal expansion in HTLV-1–infected cells compared with ACs (Figure 2D). These results are consistent with previous results showing that patients with HAM/TSP have higher HTLV-1–infected T cells with polyclonal expansions (24). HTLV-1 virus was integrated at the active transcription site of the host genome with more viral protein expression in patients with HAM/TSP (47, 48). Strong HTLV-1–specific CTL responses to HTLV-1 have been identified in the CNS as well as in peripheral blood, resulting in degranulation activity and the production of proinflammatory cytokines in the CNS (8, 49, 50). Several HLA alleles appear to be associated with predisposing or protective effects against the development of HAM/TSP (37, 51). The interaction between HTLV-1–infected cells and the host immune system is involved in the pathogenesis of HAM/TSP. While mono- or oligoclonal expansion of HTLV-1–infected T cells is a key factor in the development of ATLL, many polyclonal HTLV-1–infected T cells may elicit an inflammatory response in the host and may lead to the development of HAM/TSP.

Elimination of long-term HTLV-1–infected CD4^+^ T cells prevents the development of HTLV-1–associated disease. HTLV-1 viral proteins including Tax and HBZ drive the clonal expansion of the infected cells. However, because antigen stimulation promotes activation and expansion in CD4^+^ T cells, HTLV-1–infected T cells proliferate in response to antigen exposure. Indeed, proliferating CD4^+^ T cell responses to chronic viral antigens were identified in the HIV-1 latent reservoir (52, 53). Examination of the antigen-specific clustering by shared structural features in HTLV-1–infected people revealed that there were more clusters with a higher number of clonotypes in HTLV-1–infected cells across participants, compared with noninfected cells (Figure 5, A and B). These results suggest that antigen-driven selection or activation may be partially involved in the clonal proliferation of the HTLV-1 latent reservoir.

In conclusion, we show that the TCRβ repertoire was highly expanded in HTLV-1–infected cells. Longitudinal analysis shows that the TCR repertoire signature in HTLV-1–infected cells was conserved within each HAM/TSP patient and dominant clones were tracked over years. Expanded HTLV-1–infected clones exhibited different distributions between the CSF and peripheral blood and were enriched in the CSF of patients with HAM/TSP. These results reveal that TCR analysis of HTLV-1–infected cells detected potential pathological clones longitudinally that infiltrated the CNS. TCR repertoire analysis of HTLV-1–infected cells may provide a personalized biomarker for monitoring disease status and assessing treatment efficacy in patients with HAM/TSP. While TCR repertoire analysis may be a clinically applicable technique, the limitation of this study is the small number of participants and the limited period of observation. Each patient shows some persistent clonotypes in the repertoire during the time; however, it is not an indication per se that these are pathogenic, and difference in CSF repertoire may be due to the compartment, not only to the disease. Further research with more participants is needed to explore the association between the TCR repertoire signature in HTLV-1–infected cells and the clinical state of patients with HAM/TSP.

## Methods

### Subjects.

A total of 17 subjects, including 12 patients with HAM/TSP and 5 ACs, were enrolled in this study. Patients with HAM/TSP were diagnosed according to the World Health Organization criteria (54). Subsets of patients with HAM/TSP were used for specific studies. For analysis of paired PBMCs and CSF cells, peripheral blood and CSF samples were collected on the same day from 3 patients with HAM/TSP. Subject characteristics including sex, age, and disease status are listed in Table 1. OMDS is a clinical scale for assessing motor impairment in patients with HAM/TSP (55). The OMDS ranges from 0 to 13 in 1-unit increments that represent higher levels of disability. PBMCs were isolated from the peripheral blood by the Ficoll-Hypaque density gradient centrifugation method according to the manufacturer’s instructions and cryopreserved in liquid nitrogen until use. CSF samples were obtained by a nontraumatic lumbar puncture; subsequently, cells were collected by centrifugation at 400*g* for 10 minutes at 4°C. The CSF cells were cryopreserved in RNAlater solution (Invitrogen, Thermo Fisher Scientific).

### HTLV-1 PVL.

DNA was extracted from the PBMCs and CSF cells using the DNeasy Blood and Tissue kit (Qiagen) according to the manufacturer’s instructions. HTLV-1 PVL in the PBMCs was quantified by real-time PCR as reported previously (4). Because the amount of DNA obtained from the CSF is low, the PVL in the CSF was quantified using the following correction formula: PVL = (corrected tax copy number)/([corrected actin copy number]/2) × 100 (% copy/100 cells).

The correction formula was derived from an experiment where HTLV-1 PVL was measured in serially diluted DNA samples, and the difference was calculated between the predicted value and the actual measured value as described in Supplemental Figure 5.

### Flow sorting of CADM1^+^ populations.

Flow cytometry sorting of CADM1^+/–^CD4^+^ cells from 1 ***×*** 10^7^ PBMCs was performed on an SH800 cell sorter (Sony Biotechnology), according to the gating strategy described in Figure 1A. The antibodies used in the experiments were CD4-PC5 (Beckman Coulter, IM2636), CADM1-biotin (provided by Project for Advanced Medical Research and Development, Project Research Division, Frontier Science Research Center, University of Miyazaki, Miyazaki, Japan), and PE-streptavidin (BioLegend, 405204). The purity of CADM1^+^CD4^+^ T cells and CADM1^–^CD4^+^ T cells was 91.3%–99.5% and 83.1%–99.5%, respectively. After sorting, cells were washed in PBS, lysed in Buffer RLT (Qiagen), and stored at –80°C until use. All antibodies used for flow cytometry are provided in Supplemental Table 5.

### TCRβ library preparation and sequencing.

The sorted CADM1^+^CD4^+^ T cells, CADM1^–^CD4^+^ T cells, and CSF cells were used at 5 × 10^4^ to 5 × 10^5^ cells, 1 × 10^5^ to 5 × 10^5^ cells, and 2 × 10^4^ to 3 × 10^4^ cells, respectively, for the amplification of the TCRβ chain. Total RNA was extracted using an RNeasy Micro kit (Qiagen) following the manufacturer’s instructions. TCRβ amplification was performed according to previously described methods (20, 21). Briefly, cDNA synthesis was performed using the anchored switch 5′ rapid amplification of cDNA ends (5′RACE) PCR-based primer combined with a UMI that consists of 10 random nucleotide sequences. Subsequently, 2-stage nested PCR amplification was conducted using a pair of primers specific for the TCR constant region and the 5′RACE region, which contains a sample index and Illumina adapter sequences. TCRβ libraries were sequenced on the Illumina Miseq system with a pair-end of 150 bp.

### TCRβ repertoire analysis.

Sequence data in fastq format were processed for CDR3 extraction and VDJ gene segment alignment using MiGEC software pipeline with default parameters (56). In the postprocessing steps, we conducted further analysis using VDJtools (57) and immunarch (58), which allowed computation of basic sample statistics and visualization. Read counts, the number of clonotypes, diversity estimations, repertoire overlap analysis, and tracking of clonotypes across time points were evaluated and visualized using these software tools. Repertoire overlap was evaluated using the overlap coefficient provided by immunarch (58), which is a normalized measure of overlap similarity and defined as the size of the intersection divided by the smaller size of the 2 sets. To assess clonal expansion of T cells, we modeled the coefficient variation (CV) for each clonotype by the average number of UMIs observed across technical triplicates generated from 2 million PBMCs in the previous study (20). From inspection of this model, exponential variability in number of UMIs can be seen for those clones at inflection point on the curve of less than 8 UMIs (the cutoff value for the model with exponential was determined as log 2^3^ of read count) that was associated with a CV of about 15%. Based on this observation, TCRβ clones in each subject were classified into 1 of 3 groups: singletons (1 UMI), clones (2 ≤ UMIs < 8), and clones ≥ 8 UMIs, which were defined according to the coefficients of variation analysis for each clonotype by the average number of UMIs observed across technical triplicates (20, 21). Clustering TCR analysis was performed using the GLIPH2 algorithm (29). Significant clusters were considered based on the following parameters: final_score < 0.05 (the score is calculated by combining a specificity group score for enrichment of a motif, V-gene, CDR3 length, shared HLA among individuals, and proliferation count), number of samples ≥ 2, number of CDR3 ≥ 3. Network graphs were visualized using Cytoscape (59). TCR sequence data from normal donors were obtained from our previous study (21).

### Statistics.

Wilcoxon signed-rank test was used to compare clonal expansion and Shannon diversity between CADM1^+^CD4^+^ and CADM1^–^CD4^+^ T cells. The Mann-Whitney *U* test was used to compare clonal expansion in CADM1^+^CD4^+^ T cells between patients with HAM/TSP and ACs. Spearman’s rank correlation test was used to compare HTLV-1 PVL in PBMCs or OMDS with clonal expansion in CADM1^+^CD4^+^ T cells. All statistical analyses were performed using Prism version 9.4.0 (GraphPad software) or R version 4.0.5 (R software). *P* < 0.05 was considered statistically significant. Data are presented as median ± IQR.

### Study approval.

The study was approved by the IRB of Kagoshima University (IRB protocol no. G491). All participants provided written informed consent prior to participation, in accordance with the Declaration of Helsinki.

## Author contributions

S Nozuma designed and supervised the project, performed the experiments, analyzed the data, and wrote the paper. EM provided clinical support, analyzed the data, and wrote the paper. MT and DK analyzed the data and wrote the paper. TM provided clinical support. AY and YS provided HTS analysis. S Nakahata and KM provided flow sorting support. YEA and SJ developed the methodology and reviewed the paper. RK designed and supervised the project, analyzed the data, and wrote the manuscript. HT designed and supervised the project, analyzed the data, and wrote the manuscript.

## Supplementary Material

Supplemental data

Supplemental table 3

Supplemental table 4

## Figures and Tables

**Table 1:**
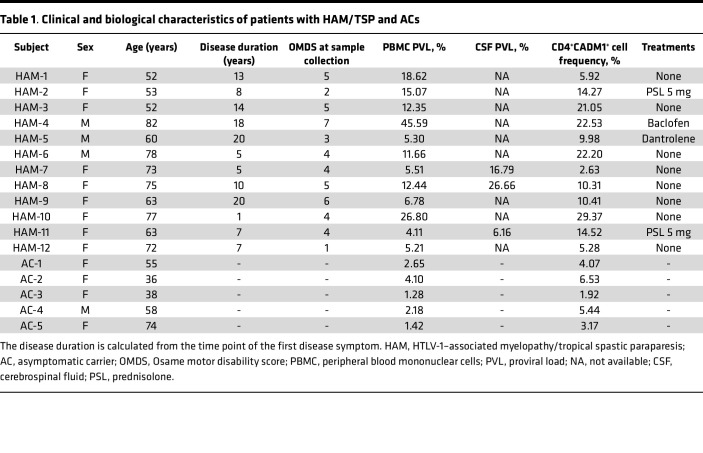
Clinical and biological characteristics of patients with HAM/TSP and ACs

**Table 2:**
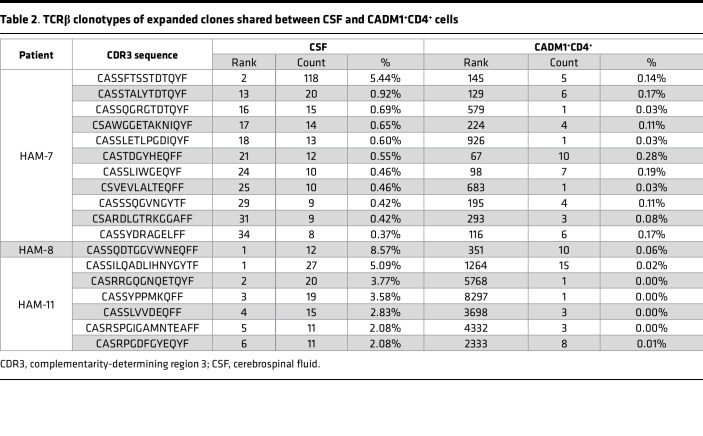
TCRβ clonotypes of expanded clones shared between CSF and CADM1^+^CD4^+^ cells

## Version 1 04/10/2023

Electronic publication
