# Supplementary material for: Identification and tracking of HTLV-1–infected T cell clones in virus-associated neurologic disease
Source: JCI Insight. 2023 Apr 10;8(7):e167422. doi: 10.1172/jci.insight.167422 (PMC10132145; doi:10.1172/jci.insight.167422)
Supplement: Supplemental data [file jciinsight-8-167422-s155.pdf]

Identification and tracking of pathological T cell clones in virus-associated neurologic disease

Satoshi Nozuma[1], Eiji Matsuura[1], Masakazu Tanaka[2], Daisuke Kodama[2], Toshio Matsuzaki[2], Akiko Yoshimura[1], Yusuke Sakiyama[1], Shingo Nakahata[3], Kazuhiro Morishita[4], Yoshimi Enose-Akahata[5], Steven Jacoboson[5], Ryuji Kubota[2], Hiroshi Takashima[1]

Supplemental materials

Supplemental figures: 5

Supplemental tables: 5

削除: 4

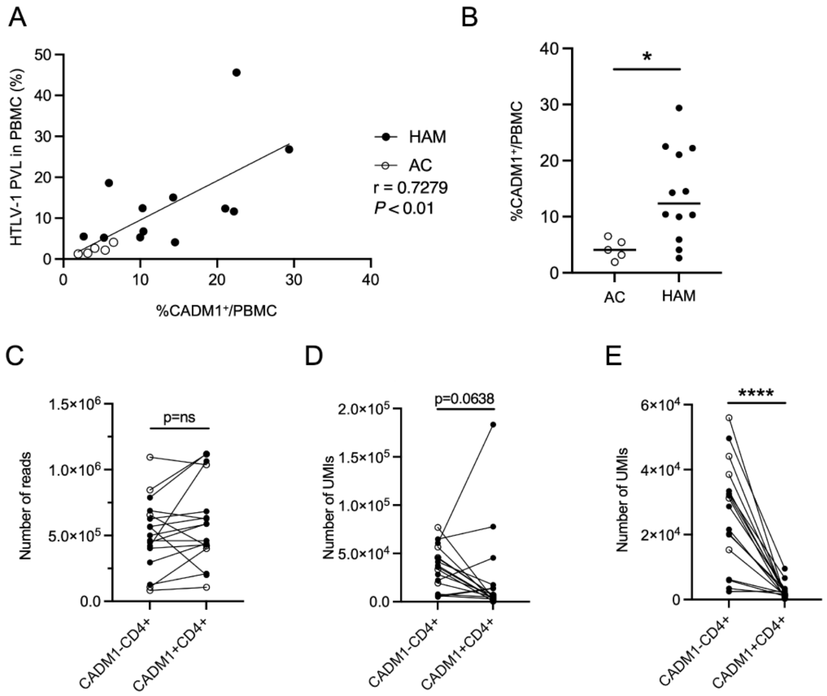

Figure S1. TCR clonal expansion and diversity of TCR β repertoire in paired CADM1$^-$CD4$^+$ and CADM1$^+$CD4$^+$ cells of HAM/TSP patients and ACs. (A) Correlation of the frequency of CADM1$^+$CD4$^+$ T cells in PBMC with HTLV-1 PVL in PBMC of HAM/TSP patients (n = 12) and ACs (n = 5) using Spearman's rank correlation test. **(B)** Comparison of the frequency of CADM1$^+$CD4$^+$ T cells in PBMCs between ACs (n = 5) and HAM/TSP patients (n = 12) using the Mann-Whitney *U* test. Comparison of the number of total sequence reads **(C)**, total unique TCR β clonotypes **(D)**, and unique TCR β clonotypes **(E)** between paired CADM1$^-$CD4$^+$ and CADM1$^+$CD4$^+$ cells of ACs (n = 5) and HAM/TSP patients (n = 12) using Wilcoxon signed-rank test. *P < 0.05, ****P <0.0001.

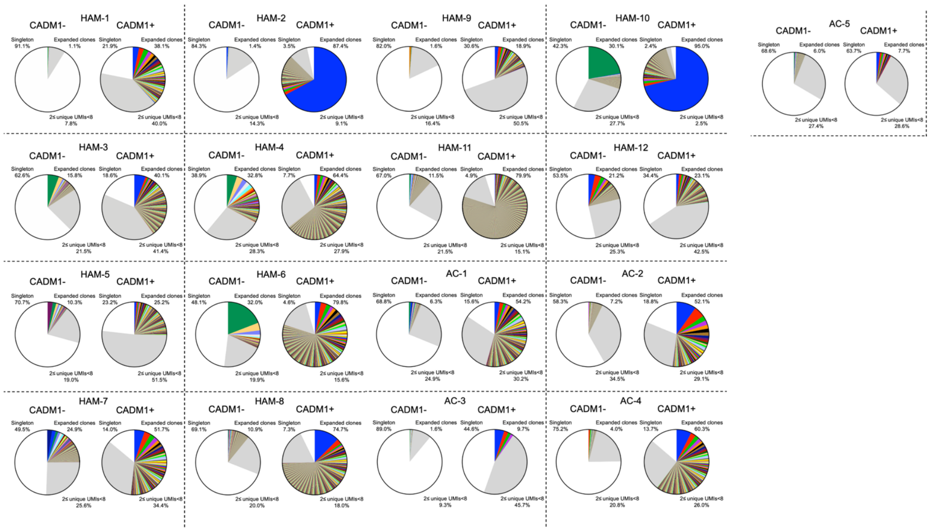

Figure S2. Analysis of T cell clonal expansion in CADM1$^-$CD4$^+$ and CADM1$^+$CD4$^+$ cells of HAM/TSP patients and ACs. Clonal expansion of TCR β repertoire was examined in paired CADM1$^-$CD4$^+$ and CADM1$^+$CD4$^+$ cells of HAM/TSP patients (n = 12) and ACs (n = 5) by using the frequencies of clones $\geq$8 unique UMIs (colored wedges), clones with 2$\leq$unique UMIs<8 (grey area) and singletons (white area). In the category of expanded clones, each wedge exhibits a unique clonotype with a defined CDR3 sequence, and the same clone shared by CADM1$^-$CD4$^+$ and CADM1$^+$CD4$^+$ cells in each individual is depicted in the same color.

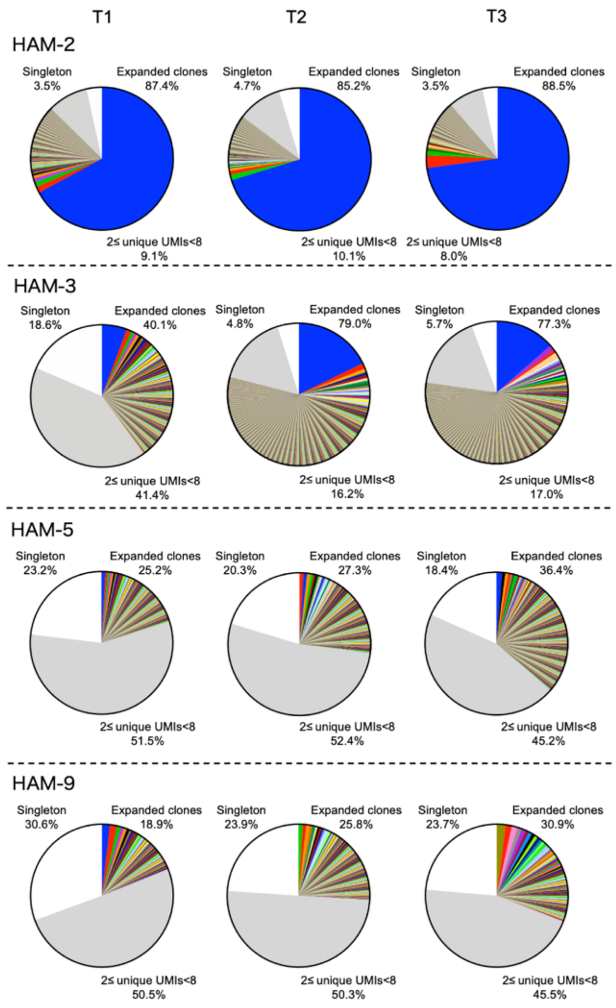

Figure S3. Longitudinal analysis of T cell clonal expansion in CADM1$^+$CD4$^+$ cells of HAM/TSP patients. T cell clonal expansion was examined in CADM1$^+$CD4$^+$ cells of HAM/TSP patients (n = 4) across 3-time points by using the frequencies of clones ≥8 unique UMIs (colored wedges), clones with 2≤unique UMIs<8 (grey area), and singletons (white area). In the category of expanded clones, each wedge exhibits a unique clonotype with a defined CDR3 sequence, and the same clone shared across 3-time points in each individual is depicted in the same color.

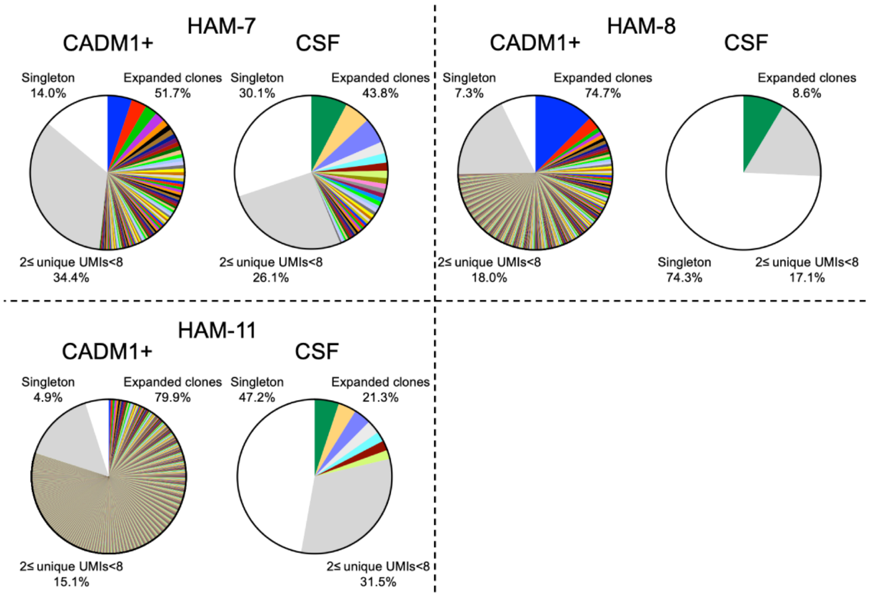

Figure S4. Analysis of T cell clonal expansion in paired CADM1^{+}CD4^{+} sorted from PBMCs and CSF cells of HAM/TSP patients. T cell clonal expansion was analyzed in paired CADM1^{+}CD4^{+} and CSF cells of HAM/TSP patients (n = 3) by using the frequencies of clones ≥8 unique UMIs (colored wedges), clones with 2≤unique UMIs<8 (grey area), and singletons (white area). In the category of expanded clones, each wedge exhibits a unique clonotype with a defined CDR3 sequence, and the identical clone shared by CADM1^{+}CD4^{+} and CSF cells in each individual is depicted in the same color.

A

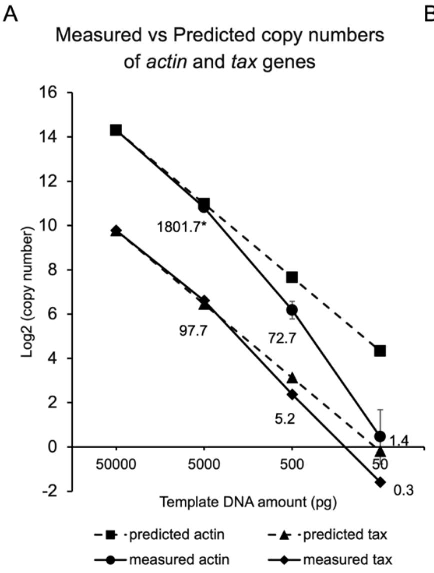

B

	Measured copy number	Correction formula
actin	1.4< actin <72.7	X = 0.581x + 4.067
	72.7< actin <1801.7	X= 0.717x + 3.223
tax	0.3< tax <5.2	Y = 0.838y + 1.136
	5.2< tax <97.7	Y = 0.785y + 1.262

(X=log2 corrected actin, x=log2 measured actin,
Y=log2 corrected tax, y=log2 measured tax)

Figure S5. Correction of low copy number of HTLV-1 proviral load. (A) Comparison between measured and predicted copy number of *actin* or *tax* genes. DNA containing 8.67 copies/100 cells of *tax* gene was serially diluted, and the copy numbers were calculated according to each standard curve. The dash and solid lines indicate predicted curves and measured curves (which were created by the calculated copy numbers), respectively. Experiments were done three times and the averages are plotted. The vertical bars indicate standard deviations and the numbers within the graph show the calculated copy numbers. The correction formulas below were obtained from this graph. According to the formulas, the copy numbers for *actin* and *tax* genes were corrected. Finally, the corrected PVL were calculated according to the following formula; PVL = (corrected *tax* copy number)/((corrected *actin* copy number)/2) x 100 (% copy/100 cells). **(B)** The correction formulas for *actin* or *tax* copy number.

Table S1. Summary of TCR β sequence data from HAM/TSP patients and ACs

Subject	Cell type	No. of total HTS sequences	No. of total UMIs	No. of unique TCR β clonotypes	% Clonal expansion	Shannon diversity
HAM-1 P	CD4+CADM1+	429,304	2871	1054	38.07%	2.77
HAM-2-T1 P	CD4+CADM1+	589,756	45438	3192	87.39%	0.09
HAM-2-T2 P	CD4+CADM1+	718,176	44591	3785	85.19%	-
HAM-2-T3 P	CD4+CADM1+	579,016	63823	4139	88.53%	-
HAM-3-T1 P	CD4+CADM1+	460,454	7388	2508	40.06%	3.73
HAM-3-T2 P	CD4+CADM1+	681,630	45350	5397	78.95%	-
HAM-3-T3 P	CD4+CADM1+	783,130	47957	6228	77.27%	-
HAM-4 P	CD4+CADM1+	439194	13739	2579	64.38%	3.47
HAM-5-T1 P	CD4+CADM1+	634,350	4832	2042	25.23%	4.45
HAM-5-T2 P	CD4+CADM1+	546303	5566	2179	27.29%	-
HAM-5-T3 P	CD4+CADM1+	285991	7610	2669	36.43%	-
HMA-6 P	CD4+CADM1+	199,264	14046	1633	79.82%	2.29
HAM-7 P	CD4+CADM1+	210,365	3,615	970	51.67%	2.28
HAM-8 P	CD4+CADM1+	626119	17353	2652	74.73%	2.05
HAM-9-T1 P	CD4+CADM1+	586530	3731	1844	18.90%	4.16
HAM-9-T2 P	CD4+CADM1+	167977	5791	2449	25.83%	-
HAM-9-T3 P	CD4+CADM1+	786670	4520	1827	30.88%	-
HAM-10 P	CD4+CADM1+	1064343	183374	6605	95.04%	0.07
HAM-11 P	CD4+CADM1+	1122406	77809	9533	79.94%	4.80
HAM-12 P	CD4+CADM1+	683624	6680	3372	23.07%	-
AC-1 P	CD4+CADM1+	106,440	5,154	1,427	54.23%	2.89
AC-2 P	CD4+CADM1+	422,411	3,482	1,045	52.10%	1.67
AC-3 P	CD4+CADM1+	400,211	610	364	9.67%	2.62
AC-4 P	CD4+CADM1+	1037231	6960	1693	60.27%	2.25
AC-5 P	CD4+CADM1+	1117595	1651	1237	7.69%	4.67
HAM-1 N	CD4+CADM1-	403,317	6288	5947	1.08%	6.01
HAM-2-T1 N	CD4+CADM1-	447,606	22053	19957	1.41%	5.94
HAM-2-T2 N	CD4+CADM1-	667,407	28593	24912	2.24%	-
HAM-2-T3 N	CD4+CADM1-	567,542	21523	19045	1.96%	-
HAM-3-T1 N	CD4+CADM1-	460,372	28214	20243	15.85%	4.55
HAM-3-T2 N	CD4+CADM1-	210,367	39872	24783	20.85%	-
HAM-3-T3 N	CD4+CADM1-	878,477	57339	41031	14.21%	-
HAM-4 N	CD4+CADM1-	295653	5012	2486	32.80%	3.15
HAM-5-T1 N	CD4+CADM1-	568,668	7824	6181	10.30%	4.90

HAM-5-T2 N	CD4+CADM1-	503046	8209	6492	7.49%	-
HAM-5-T3 N	CD4+CADM1-	700152	29521	24072	6.17%	-
HAM-6 N	CD4+CADM1-	564,393	6081	3418	31.95%	1.72
HAM-7 N	CD4+CADM1-	126,390	35,903	21,592	24.89%	4.68
HAM-8 N	CD4+CADM1-	688084	41310	32025	10.93%	5.87
HAM-9-T1 N	CD4+CADM1-	500582	37360	33438	1.59%	5.96
HAM-9-T2 N	CD4+CADM1-	563513	42011	37631	2.40%	-
HAM-9-T3 N	CD4+CADM1-	640134	37976	31830	4.63%	-
HAM-10 N	CD4+CADM1-	419785	60513	32504	30.05%	1.94
HAM-11 N	CD4+CADM1-	788189	64714	49613	11.53%	5.85
HAM-12 N	CD4+CADM1-	626428	45070	28681	21.24%	-
AC-1 N	CD4+CADM1-	82,768	19,280	15,341	6.25%	5.78
AC-2 N	CD4+CADM1-	655,040	76,997	55,996	7.21%	5.97
AC-3 N	CD4+CADM1-	107,902	33,465	31,222	1.65%	6.05
AC-4 N	CD4+CADM1-	1094684	45719	38549	3.99%	5.92
AC-5 N	CD4+CADM1-	845316	56508	44121	6.01%	5.92
HAM7-CSF	CSF	212,824	2,168	892	43.82%	-
HAM8-CSF	CSF	373898	140	116	8.57%	-
HAM-11 CSF	CSF	359874	530	313	21.32%	-

Shannon diversity is estimated by normalizing the number of unique T cell clonotypes via resampling to the size of the smallest data set of sorted $CD4^{+}CADM1^{+}$ or $CD4^{+}CADM1^{-}$ cells

Table S2. TCR β clonotypes of clones shared between CSF and CADM1⁺CD4⁺ cells

Patient	CDR3 sequence	CSF			CADM1		
		Rank	Count	%	Rank	Count	%
HAM-7	CASSFTSSTDTQYF	2	118	5.44%	145	5	0.14%
	CASSTALYTDTQYF	13	20	0.92%	129	6	0.17%
	CASSQGRGTDTQYF	16	15	0.69%	579	1	0.03%
	CSAWGGETAKNIQYF	17	14	0.65%	224	4	0.11%
	CASSLETLPGDIQYF	18	13	0.60%	926	1	0.03%
	CASTDGYHEQFF	21	12	0.55%	67	10	0.28%
	CASSLIWGEQYF	24	10	0.46%	98	7	0.19%
	CSVEVLALTEQFF	25	10	0.46%	683	1	0.03%
	CASSSQGVNGYTF	29	9	0.42%	195	4	0.11%
	CSARDLGTRKGGAFF	31	9	0.42%	293	3	0.08%
	CASSYDRAGELFF	34	8	0.37%	116	6	0.17%
	CASSPGTGRGNTIYF	39	7	0.32%	675	1	0.03%
	CSARGFGNQPQHF	40	7	0.32%	208	4	0.11%
	CASSSHGDTQYF	41	7	0.32%	8	34	0.94%
	CSAWEDSNQPQHF	42	6	0.28%	19	24	0.66%
	CSAIGLNYGYTF	45	6	0.28%	668	1	0.03%
	CSAGDPNQPQHF	47	6	0.28%	724	1	0.03%
	CSARGALTGYGYTF	49	5	0.23%	173	4	0.11%
	CASSLGPSKPQHF	52	5	0.23%	43	15	0.41%
	CSARVSGSLGANVLTF	54	5	0.23%	121	6	0.17%
	CASSLGRETQYF	55	5	0.23%	716	1	0.03%
	CASSLGGDSSNQPQHF	56	5	0.23%	7	40	1.11%
	CASSLVRGNNEKLFF	58	5	0.23%	40	16	0.44%
	CASSLTPSSSYNEQFF	60	4	0.18%	113	6	0.17%
	CASSTGGAGGTDTQYF	61	4	0.18%	589	1	0.03%
	CASSVRKTGTSNTGELFF	63	4	0.18%	117	6	0.17%
	CASSEYKEIGELFF	67	4	0.18%	6	40	1.11%
	CASRIQGAAGELFF	68	4	0.18%	694	1	0.03%
	CASTALGRADEQFF	69	4	0.18%	3	92	2.54%
	CASSHGVAGAVGQETQYF	79	3	0.14%	334	2	0.06%
	CASSLEGDTQYF	82	3	0.14%	24	21	0.58%

CASNGGPNTGELFF	87	3	0.14%	194	4	0.11%
CASSSSGGEYNEQFF	92	3	0.14%	153	5	0.14%
CASTPGRGNSPLHF	95	3	0.14%	203	4	0.11%
CASSFNSGNSGGHWETQYF	96	3	0.14%	403	2	0.06%
CSELGLSSYEQYF	106	3	0.14%	885	1	0.03%
CASSSSPPDRVMGSEAFF	108	3	0.14%	446	2	0.06%
CASSEKGRGFEQYF	111	3	0.14%	908	1	0.03%
CASSPEESSPEAFF	122	2	0.09%	476	1	0.03%
CASSQGQGSDSPLHF	125	2	0.09%	135	5	0.14%
CASSLRAQRQPQHF	126	2	0.09%	231	3	0.08%
CASIPTTPQNTEAFF	131	2	0.09%	506	1	0.03%
CASSPSRRENIQYF	135	2	0.09%	332	2	0.06%
CSASGGYEQYF	137	2	0.09%	335	2	0.06%
CSVKGDTGELFF	148	2	0.09%	26	20	0.55%
CASSLRDRNSPLHF	160	2	0.09%	645	1	0.03%
CASSRGQGYPYNEQFF	161	2	0.09%	671	1	0.03%
CSATRTGHLNTEAFF	179	2	0.09%	102	7	0.19%
CASSFSLSYEQYF	193	2	0.09%	809	1	0.03%
CASSRRLSGGETQYF	194	2	0.09%	819	1	0.03%
CASSVGQGWNEQFF	197	2	0.09%	420	2	0.06%
CASSPTVNTEAFF	199	2	0.09%	90	8	0.22%
CSARPNTEAFF	219	2	0.09%	914	1	0.03%
CASSLNSYGADTQYF	237	2	0.09%	53	13	0.36%
CASSSRAGGGREQFF	238	2	0.09%	134	6	0.17%
CASSNAITEAFF	245	1	0.05%	311	2	0.06%
CASSDPGGQETQYF	259	1	0.05%	480	1	0.03%
CASRGGDEQFF	262	1	0.05%	230	3	0.08%
CASSYSMKVNTEAFF	269	1	0.05%	171	4	0.11%
CASRRSYNSPLHF	278	1	0.05%	316	2	0.06%
CASSYSSPLNTEAFF	303	1	0.05%	502	1	0.03%
CASSQVGRTGNEKLFF	304	1	0.05%	321	2	0.06%
CASTETSRETQYF	309	1	0.05%	176	4	0.11%
CASSSTGGINEKLFF	312	1	0.05%	513	1	0.03%
CASRPMGSNQPQHF	316	1	0.05%	514	1	0.03%

CASSWEGAKADTQYF	333	1	0.05%	29	17	0.47%
CASSPGQGASNEQFF	365	1	0.05%	560	1	0.03%
CASRTSDRLYGYTF	391	1	0.05%	63	10	0.28%
CSAGYSNQPQHF	392	1	0.05%	184	4	0.11%
CSATYVGRLNTEAFF	395	1	0.05%	586	1	0.03%
CATSDGQTFAGELFF	396	1	0.05%	95	7	0.19%
CASSRGQAVRDNEQFF	407	1	0.05%	596	1	0.03%
CASSVAGGPEETQYF	427	1	0.05%	31	17	0.47%
CSGLTPGFGGHGGYTF	436	1	0.05%	630	1	0.03%
CASSQGGEYGYTF	462	1	0.05%	366	2	0.06%
CSAREAGLLNTEAFF	465	1	0.05%	367	2	0.06%
CASSPERGGVETQYF	479	1	0.05%	73	9	0.25%
CSAQGQEGEQFF	482	1	0.05%	147	5	0.14%
CASSEIIETQYF	489	1	0.05%	676	1	0.03%
CASSLTGTKGEAFF	514	1	0.05%	700	1	0.03%
CASSSDWSSTDTQYF	545	1	0.05%	393	2	0.06%
CASSQDPGTNQPQHF	571	1	0.05%	198	4	0.11%
CSASLPGGGGYEQYF	576	1	0.05%	199	4	0.11%
CSARRDRVSGGTEAFF	587	1	0.05%	771	1	0.03%
CASSYQINTEAFF	590	1	0.05%	772	1	0.03%
CSARAQGQGLHTQYF	594	1	0.05%	775	1	0.03%
CASSISGGPTDTQYF	624	1	0.05%	282	3	0.08%
CASSSRGGFYEQYF	626	1	0.05%	158	5	0.14%
CATGVQGNSPLHF	636	1	0.05%	159	5	0.14%
CASTSEDEQFF	651	1	0.05%	286	3	0.08%
CASSVAPQNNSPLHF	679	1	0.05%	127	6	0.17%
CASSLAGQNEKLFF	688	1	0.05%	419	2	0.06%
CASSRDNHNYGYTF	704	1	0.05%	427	2	0.06%
CASSVRGGSTDTQYF	715	1	0.05%	431	2	0.06%
CASSSTGVYGYTF	725	1	0.05%	858	1	0.03%
CASSDSTTGPETQYF	729	1	0.05%	435	2	0.06%
CSARDNTGELFF	745	1	0.05%	441	2	0.06%
CSVVGEGDQPQHF	748	1	0.05%	877	1	0.03%
CASRLSARGTTYEQYF	755	1	0.05%	104	7	0.19%

	CASGLHQGGGNQPQHF	761	1	0.05%	884	1	0.03%
	CASSFGLGRQNEQFF	781	1	0.05%	892	1	0.03%
	CATSDGVAGAFDVGTQYF	797	1	0.05%	301	3	0.08%
	CASSEVTDSVEAFF	803	1	0.05%	222	4	0.11%
	CASSEVGGYTF	807	1	0.05%	910	1	0.03%
	CASSPPGGAAFF	829	1	0.05%	132	6	0.17%
	CASSVRGMNTEAFF	864	1	0.05%	462	2	0.06%
	CASSSRTAASIQYF	888	1	0.05%	465	2	0.06%
	CSATQAGRRNEQYF	890	1	0.05%	227	4	0.11%
HAM-8	CASSQDTGGVWNEQFF	1	12	8.57%	351	10	0.06%
	CASSLAGRYNEQFF	5	2	1.43%	94	29	0.17%
	CASSLSNSGNTGELFF	6	2	1.43%	384	9	0.05%
	CASSQGIGQSYEQYF	8	2	1.43%	210	16	0.09%
	CASSSDRVHPEQYF	10	2	1.43%	344	10	0.06%
	CASSPLGSDTQYF	13	1	0.71%	762	4	0.02%
	CASSTGQSNSPLHF	22	1	0.71%	189	17	0.10%
	CASSIRTSGLQGHEQYF	37	1	0.71%	813	4	0.02%
	CGAKQPGQNTGELFF	47	1	0.71%	50	46	0.27%
	CSASPTGTGTDTQYF	48	1	0.71%	1161	2	0.01%
	CSASFGVTSGNEQFF	51	1	0.71%	177	18	0.10%
	CASSLEVGGHQHF	54	1	0.71%	1659	1	0.01%
	CASSQRSRGNTGELFF	56	1	0.71%	552	6	0.03%
	CSASARSGSYEQYF	59	1	0.71%	1406	1	0.01%
	CASSPHDREEAFF	61	1	0.71%	423	8	0.05%
	CSASGSIHEQFF	63	1	0.71%	8	110	0.63%
	CSALEPGLAGETQYF	64	1	0.71%	208	16	0.09%
	CASSATSGTWETQYF	69	1	0.71%	242	14	0.08%
	CASSLTGESYEQYF	73	1	0.71%	13	90	0.52%
	CASSLRNFANTGELFF	76	1	0.71%	155	19	0.11%
	CSERNLEGTQYF	79	1	0.71%	329	11	0.06%
	CASSKLGETQYF	80	1	0.71%	312	11	0.06%
	CASSLLGGEYNEQFF	85	1	0.71%	193	17	0.10%
	CSATDRGLNQPQHF	90	1	0.71%	711	4	0.02%
HAM-11	CASSILQADLIHNYGYTF	1	27	5.09%	1264	15	0.02%

CASRRGQGNQETQYF	2	20	3.77%	5768	1	0.00%
CASSYPPMKQFF	3	19	3.58%	8297	1	0.00%
CASSLVVDEQFF	4	15	2.83%	3698	3	0.00%
CASRSPGIGAMNTEAFF	5	11	2.08%	4332	3	0.00%
CASRPGDFGYEQYF	6	11	2.08%	2333	8	0.01%
CASSSLAGEETQYF	8	7	1.32%	4	314	0.40%
CASSHPSNYGYTF	9	7	1.32%	3518	4	0.01%
CASSPTSGYEQYF	10	7	1.32%	547	30	0.04%
CASSFRSLWAGELFF	11	6	1.13%	4097	3	0.00%
CSARGASGRTYEQYF	14	5	0.94%	25	172	0.22%
CASSSVTGAGSNQPQHF	15	5	0.94%	335	42	0.05%
CASSAGTSDRTDTQYF	16	4	0.75%	4618	2	0.00%
CASSQLSGGGATEAFF	17	4	0.75%	936	20	0.03%
CSAREQGRAKNIQYF	18	4	0.75%	399	37	0.05%
CSVLVRDLAKNIQYF	19	4	0.75%	8770	1	0.00%
CSASRQGLSQPQHF	21	3	0.57%	570	29	0.04%
CASSTRGGAEAFF	23	3	0.57%	511	32	0.04%
CASSSAQLYSGVHSGANVLTF	25	3	0.57%	1393	14	0.02%
CASSLEGGQGSNQPQHF	26	3	0.57%	4192	3	0.00%
CAISEGGQQNVEAFF	27	3	0.57%	3685	3	0.00%
CASSRGTGLQNSPLHF	28	3	0.57%	885	21	0.03%
CASSLDRRMNTEAFF	29	3	0.57%	2262	8	0.01%
CASSLLKTASSYEQYF	30	3	0.57%	491	32	0.04%
CASSLDPDREVCF	31	3	0.57%	1507	13	0.02%
CASSYVREYEQYF	32	3	0.57%	1420	14	0.02%
CASSPTGGLETQYF	33	2	0.38%	356	40	0.05%
CASSLTRVEQFF	35	2	0.38%	243	52	0.07%
CASSQGRRLEQFF	36	2	0.38%	206	56	0.07%
CASSLKTSGSRETQYF	37	2	0.38%	106	81	0.10%
CASSQLADANTGELFF	38	2	0.38%	1093	17	0.02%
CASSLGTSGMVDEQYF	39	2	0.38%	325	43	0.06%
CSAGQGFNNEQFF	41	2	0.38%	11	244	0.31%
CAISESSNPTDTQYF	42	2	0.38%	908	20	0.03%
CASSQGDSNQPQHF	43	2	0.38%	255	50	0.06%

CASSDDRVGEQFF	44	2	0.38%	8734	1	0.00%
CASSLVGTGRNIQYF	45	2	0.38%	235	53	0.07%
CASSAASGRTETQYF	46	2	0.38%	234	53	0.07%
CASSPGTPDEQYF	47	2	0.38%	6082	1	0.00%
CASSSGVTLIAGELFF	48	2	0.38%	1303	15	0.02%
CASKRRDGLLSYNEQFF	49	2	0.38%	2880	6	0.01%
CASSSGLAGTVSYNEQFF	52	2	0.38%	4119	3	0.00%
CASSLTGPNSPLHF	53	2	0.38%	3786	3	0.00%
CASMGGRGSGANVLTF	56	2	0.38%	3084	5	0.01%
CSPLGEGADTQYF	57	2	0.38%	2	322	0.41%
CASSPRTGSTDTQYF	58	2	0.38%	8050	1	0.00%
CASSQRTVGLYEQYF	59	2	0.38%	5390	2	0.00%
CATSDGDTQYF	60	2	0.38%	2328	8	0.01%
CASSPLQGEQYF	62	2	0.38%	4045	3	0.00%
CASSSIRTVYNEQFF	63	2	0.38%	1069	18	0.02%
CASASPRLRGDTIYF	65	1	0.19%	1	327	0.42%
CASSQDWRASYEQYF	69	1	0.19%	912	20	0.03%
CASSVQGSTGELFF	70	1	0.19%	1466	13	0.02%
CASSQAGPKTKTTSTDTQYF	72	1	0.19%	3965	3	0.00%
CSVETHGYTF	73	1	0.19%	1485	13	0.02%
CASSLDTPDTQYF	75	1	0.19%	1788	11	0.01%
CASSETGGGDQPQHF	78	1	0.19%	1087	17	0.02%
CSASPLGAGRYNEQFF	81	1	0.19%	16	211	0.27%
CASSQGHTGELFF	82	1	0.19%	539	30	0.04%
CAISEYSGRVYEQYF	83	1	0.19%	407	37	0.05%
CASRRGHSGNTIYF	84	1	0.19%	6896	1	0.00%
CASSENRGDTQYF	86	1	0.19%	1228	16	0.02%
CASSLLRSGGAADTQYF	88	1	0.19%	268	49	0.06%
CASSSRGNGRLQEQFF	91	1	0.19%	8327	1	0.00%
CSARDSSASGKETQYF	93	1	0.19%	155	67	0.09%
CATSEGTGRTDTQYF	98	1	0.19%	242	52	0.07%
CASSLNRNQPQHF	99	1	0.19%	689	25	0.03%
CSAAALGYRNTEAFF	102	1	0.19%	33	151	0.19%
CSVTGGASGRAEQYF	104	1	0.19%	58	117	0.15%

CASSLGPTTVTGLDGYTF	106	1	0.19%	141	70	0.09%
CASSLPGRAGGELFF	107	1	0.19%	1520	13	0.02%
CASSLEAGGGVREQYF	108	1	0.19%	6909	1	0.00%
CASSLGTSGRYEQYF	109	1	0.19%	7144	1	0.00%
CSAGGGISNSPLHF	110	1	0.19%	1617	12	0.02%
CSARGEGNYGYTF	111	1	0.19%	3774	3	0.00%
CASSVINEQFF	113	1	0.19%	458	34	0.04%
CASSQDMGQGYEQYF	120	1	0.19%	8455	1	0.00%
CASKGPGGTDTQYF	122	1	0.19%	7641	1	0.00%
CASSLQLAGSDTQYF	123	1	0.19%	3989	3	0.00%
CASSFPGRGAGELFF	124	1	0.19%	2424	7	0.01%
CSVEGPTTNEKLFF	125	1	0.19%	125	74	0.10%
CASSPPGRNEKLFF	131	1	0.19%	4923	2	0.00%
CASSESGTRGANVLTF	135	1	0.19%	4795	2	0.00%
CASSFSPGATYEQYF	136	1	0.19%	180	61	0.08%
CASSQQGGVSYNEQFF	140	1	0.19%	785	23	0.03%
CASSVAPRSLDTQYF	141	1	0.19%	853	21	0.03%
CASSPLENTEAFF	142	1	0.19%	314	44	0.06%
CASSLGDSRSYEQYF	144	1	0.19%	237	53	0.07%
CASSLPRFGATEAFF	145	1	0.19%	101	81	0.10%
CASSHREETQYF	149	1	0.19%	115	77	0.10%
CASSESENTEAFF	153	1	0.19%	228	53	0.07%
CASSSGGWVTEAFF	156	1	0.19%	179	61	0.08%
CASSPLPGTGGYEQFF	157	1	0.19%	1001	19	0.02%
CASSSDRVQYF	159	1	0.19%	4529	2	0.00%
CSAVKTRPYEQYF	163	1	0.19%	2585	6	0.01%
CASSATGTLAKNIQYF	164	1	0.19%	884	21	0.03%
CSASTGYGYTF	169	1	0.19%	3639	4	0.01%
CASSQDVGPLHF	171	1	0.19%	6930	1	0.00%
CASSNRGGYTF	172	1	0.19%	3840	3	0.00%
CSARPLAGGPDTQYF	175	1	0.19%	67	108	0.14%
CASSWTGADTGELFF	176	1	0.19%	38	141	0.18%
CATSDVTGIGYNEQFF	177	1	0.19%	181	61	0.08%
CASSRGTSGRGPQETQYF	178	1	0.19%	4075	3	0.00%

CASSLRVNTEAFF	179	1	0.19%	9469	1	0.00%
CASSPREGWTDTQYF	181	1	0.19%	2104	9	0.01%
CATSGSSRHTQYF	182	1	0.19%	1861	10	0.01%
CASSLGGRSYYEQYF	185	1	0.19%	479	33	0.04%
CASSGGDTQYF	188	1	0.19%	56	119	0.15%
CASSPPGSQETQYF	189	1	0.19%	3228	5	0.01%
CSAPPRGRGSDTQYF	190	1	0.19%	640	27	0.03%
CASSYSRTEAFF	193	1	0.19%	7571	1	0.00%
CASSYSGTQETQYF	203	1	0.19%	139	71	0.09%
CASSSGQSNQPQHF	204	1	0.19%	302	46	0.06%
CASSPVAGGSYNEQFF	205	1	0.19%	771	23	0.03%
CSARVPSVANTGELFF	207	1	0.19%	1523	13	0.02%
CSGRRGEETQYF	208	1	0.19%	289	47	0.06%
CSAWTSGRGTDTQYF	210	1	0.19%	170	63	0.08%
CSARDQITGRATNEKLFF	215	1	0.19%	440	35	0.04%
CASRAGGGGYTF	221	1	0.19%	2211	8	0.01%
CSARATGGARTEAFF	222	1	0.19%	398	37	0.05%
CASSNRTTYEQYF	223	1	0.19%	1949	10	0.01%
CASSPGSSGGQETQYF	224	1	0.19%	624	27	0.03%
CSAATGVDQPQHF	225	1	0.19%	4970	2	0.00%
CASSTGTGELFF	227	1	0.19%	1895	10	0.01%
CSAPTPGGSGEETQYF	229	1	0.19%	1328	15	0.02%
CASSRPGGDYEQYF	233	1	0.19%	466	34	0.04%
CASSVTSGYEQYF	234	1	0.19%	716	25	0.03%
CASRQGRSEAFF	237	1	0.19%	347	41	0.05%
CASSREMNTEAFF	238	1	0.19%	2336	8	0.01%
CASSHNWLAGNEQFF	240	1	0.19%	294	47	0.06%
CSSGERNAEAFF	241	1	0.19%	784	23	0.03%
CASSSRPDSNQPQHF	243	1	0.19%	5639	2	0.00%
CASSVTALSGGTGELFF	244	1	0.19%	210	55	0.07%
CASSYLPNEKLFF	245	1	0.19%	1939	10	0.01%
CAISASRTLGNYEQYF	247	1	0.19%	63	110	0.14%
CASSSGQGETDTQYF	250	1	0.19%	372	39	0.05%
CSASWGQGSYNEQFF	251	1	0.19%	619	28	0.04%

CASSLVVTGVAEQYF	253	1	0.19%	8282	1	0.00%
CASSPGQVRTDTQYF	254	1	0.19%	1534	13	0.02%
CSARRGRANEQFF	257	1	0.19%	7359	1	0.00%
CASSLAGAAETQYF	258	1	0.19%	7360	1	0.00%
CASSLPREGGYTF	262	1	0.19%	3249	4	0.01%
CAIRGDDYSYEQYF	266	1	0.19%	3734	3	0.00%
CASSPRDRGTDTQYF	268	1	0.19%	2798	6	0.01%
CASSHGWGQWLFEQYF	269	1	0.19%	590	29	0.04%
CSAREYRGLNTEAFF	271	1	0.19%	297	46	0.06%
CASSPSKFSNQPQHF	272	1	0.19%	22	179	0.23%
CSAGAGAIRETQYF	273	1	0.19%	409	37	0.05%
CAISEPQGLVNSNEQFF	274	1	0.19%	211	55	0.07%
CASGSSGTDNEQFF	275	1	0.19%	857	21	0.03%
CASSPGTGGNQPQHF	276	1	0.19%	366	39	0.05%
CASSLDILNEQFF	278	1	0.19%	18	188	0.24%
CASSLATQGVSLRASEAFF	279	1	0.19%	323	43	0.06%
CASSLTSQQYF	280	1	0.19%	1971	9	0.01%
CSARVLGTQYF	281	1	0.19%	35	150	0.19%
CSARSGDRADSPLHF	283	1	0.19%	153	68	0.09%
CASSRDLVSYNEQFF	284	1	0.19%	424	36	0.05%
CASSPDFITDTQYF	287	1	0.19%	463	34	0.04%
CASSTQLGAAYEQYF	289	1	0.19%	1225	16	0.02%
CASSFRAGGSGELFF	290	1	0.19%	17	197	0.25%
CATGQGTQETQYF	291	1	0.19%	290	47	0.06%
CASSRNRGRNSPLHF	292	1	0.19%	3295	4	0.01%
CASSHRGRGTEAFF	295	1	0.19%	1495	13	0.02%
CASSSPQGSFTDTQYF	296	1	0.19%	4472	2	0.00%
CSARDFSGGFDNEQYF	298	1	0.19%	2698	6	0.01%
CASSLGRAGGTQYF	300	1	0.19%	3961	3	0.00%
CASKLDYNEQFF	303	1	0.19%	208	56	0.07%
CASSGGGGSNYGYTF	304	1	0.19%	2415	7	0.01%
CSAREAGTIQGGETQYF	305	1	0.19%	876	21	0.03%
CASSLTTGTGREQYF	309	1	0.19%	4163	3	0.00%
CASSLASGRETQYF	311	1	0.19%	692	25	0.03%

CDR3, complementary determining region 3; CSF, cerebrospinal fluid

Table S5. List of antibodies for flow cytometry sorting

Antibody	Clone	Catalog number	Manufacturer
CD4	13B8.2	IM2636	Beckman Coulter
CADM1	-	Custom antibody	Provided by Dr. Morishita (Ref: J Clin Exp Hematop. 2012;52(1):17-22.)
Streptavidin	-	405204	Biolegend
